# Metabolic benefits of inhibition of p38α in white adipose tissue in obesity

**DOI:** 10.1371/journal.pbio.2004225

**Published:** 2018-05-11

**Authors:** Shengjie Zhang, Hongchao Cao, Yan Li, Yanyan Jing, Shengnan Liu, Cheng Ye, Hui Wang, Shuxian Yu, Chengyuan Peng, Lijian Hui, Yu-cheng Wang, Haibing Zhang, Feifan Guo, Qiwei Zhai, Hui Wang, Ruimin Huang, Ling Zhang, Jingjing Jiang, Wei Liu, Hao Ying

**Affiliations:** 1 CAS Key laboratory of nutrition, metabolism and food safety, Shanghai Institutes for Biological Sciences, University of Chinese Academy of Sciences, Chinese Academy of Sciences, Shanghai, China; 2 Shanghai Institute of Materia Medica, Chinese Academy of Sciences, Shanghai, China; 3 Laboratory of Molecular Cell Biology, Institute of Biochemistry and Cell Biology, Shanghai Institutes for Biological Sciences, Chinese Academy of Sciences, Shanghai, China; 4 Shanghai Xuhui Central Hospital, Shanghai Clinical Center, Chinese Academy of Sciences, Shanghai, China; 5 Key Laboratory of Food Safety Risk Assessment, Ministry of Health, Beijing, China; 6 Department of Head and Neck Surgery, Fudan University Cancer Center and Department of Oncology, Fudan University, Shanghai Medical College, Shanghai, China; 7 Department of Endocrinology and Metabolism, Zhongshan Hospital, Fudan University, Shanghai, China; Harvard School of Public Health, United States of America

## Abstract

p38 has long been known as a central mediator of protein kinase A (PKA) signaling in brown adipocytes, which positively regulate the transcription of uncoupling protein 1 (UCP-1). However, the physiological role of p38 in adipose tissues, especially the white adipose tissue (WAT), is largely unknown. Here, we show that mice lacking p38α in adipose tissues display a lean phenotype, improved metabolism, and resistance to diet-induced obesity. Surprisingly, ablation of p38α causes minimal effects on brown adipose tissue (BAT) in adult mice, as evident from undetectable changes in UCP-1 expression, mitochondrial function, body temperature (BT), and energy expenditure. In contrast, genetic ablation of p38α in adipose tissues not only markedly facilitates the browning in WAT upon cold stress but also prevents diet-induced obesity. Consistently, pharmaceutical inhibition of p38α remarkably enhances the browning of WAT and has metabolic benefits. Furthermore, our data suggest that p38α deficiency promotes white-to-beige adipocyte reprogramming in a cell-autonomous manner. Mechanistically, inhibition of p38α stimulates the UCP-1 transcription through PKA and its downstream cAMP-response element binding protein (CREB), which form a positive feedback loop that functions to reinforce the white-to-beige phenotypic switch during cold exposure. Together, our study reveals that inhibition of p38α is able to promote WAT browning and confer metabolic benefits. Our study also indicates that p38α in WAT represents an exciting pharmacological target to combat obesity and metabolic diseases.

## Author summary

The functional brown adipose tissue (BAT) identified in human adults consists of not only classic brown adipocytes but also brown-like adipocytes (beige adipocytes), both of which are important for energy homeostasis. Due to the same ability to convert fat into heat as brown adipocytes, beige adipocytes have been considered as a novel pharmacological target to combat obesity. Growing evidence suggests that promoting the development and formation of beige adipocytes in white adipose tissue (WAT), also called the browning of WAT, is able to prevent diet-induced obesity and improve metabolism in rodents. Thus, understanding the molecular basis for the regulation of browning in WAT may help us to develop new strategies to counteract obesity and metabolic diseases. In this study, adipocyte-specific p38α knockout (Fp38αKO) mice are generated that display a lean phenotype, improved metabolism, and resistance to diet-induced obesity. Interestingly, we found that adipocyte p38α deficiency facilitates the browning in WAT. Then, we show that pharmaceutical inhibition of p38α enhances the browning in WAT and has metabolic benefits. We propose that inhibiting p38α in WAT, possibly combined with cold exposure, could constitute an exciting pharmacological target to combat obesity and metabolic diseases.

## Introduction

White adipose tissue (WAT) and brown adipose tissue (BAT) are two major types of adipose tissues, which play different physiological roles in whole-body energy homeostasis [1,2]. The main function of WAT is to store excess energy as triglycerides (TGs) for utilization during nutrient shortage and to produce bioactive adipokines—such as leptin, adiponectin, and resistin, which take part in glucose and lipid metabolism [3]—while BAT dissipates the chemical energy stored in TGs as heat to preserve core temperature through uncoupling of fatty acid oxidation from ATP production by uncoupling protein 1 (UCP-1) during hypothermia [4]. BAT was thought to function primarily in rodents and in newborn babies until functional BAT was discovered in adult humans [5,6]. The BAT identified in human adults might consist of not only classic brown adipocytes but also brown-like adipocytes (beige adipocytes). Similar to classic brown adipocytes, beige adipocytes display multilocular lipid droplet morphology, have high mitochondrial content, and express UCP-1, although they differ from classic brown adipocytes in their origin and molecular identity [7,8].

It has been indicated that the beige adipocytes interspersed among white adipocytes in rodents are able to alleviate cold stress to restore thermal homeostasis [8]. Due to the same ability to convert fat into heat through uncoupled respiration as brown adipocyte, beige adipocyte has been also considered an attractive target to promote weight loss. Indeed, promoting the development and formation of beige adipocytes in WAT, also called the browning of WAT, increases energy expenditure, prevents diet-induced obesity, and improves glucose metabolism in rodents [9,10], while suppressing WAT browning leads to obesity and insulin resistance [11,12]. It is worth noting that beige adipocytes have been shown to contribute to systemic energy handling even at room temperature (RT) [13]. Given that beige and brown adipocytes have many distinguishing characteristics [14], it is probable that the regulation of the thermogenic program differs in beige and brown adipocytes, which has yet to be studied.

p38 mitogen-activated protein kinases (MAPKs) are key mediators in cellular responses to extracellular stimuli. p38 MAPKs play critical roles in a wide variety of cellular processes such as proliferation, differentiation, regeneration, and metabolism [15–19]. The p38 family of proline-directed serine/threonine kinases has 4 members (p38α, β, γ, and δ), each encoded by individual genes. p38α is highly abundant in most cell types, while p38γ and p38δ have more restricted expression patterns [20,21]. It has been proposed that p38 family members function in a cell context–specific and cell type–specific manner to integrate signals that affect cellular processes [22,23]. p38 has long been known as a central mediator of cAMP/protein kinase A (PKA) signaling, which positively regulates the transcription of UCP-1 in brown adipocytes by phosphorylating activating transcription factor 2 (ATF2) directly, a member of the cAMP-response element binding protein (CREB)/ATF family of transcription factors [17,24,25]. The in vitro effect of p38 on the thermogenic program in brown adipocytes has been well established [17,24–26]; however, the physiologic role of p38 during cold exposure has never been validated by employing a loss-of-function genetic approach in mice. Moreover, whether cAMP/PKA/p38/ATF2 cascade plays a similar role in beige adipocytes is largely unknown.

Here, we show that mice lacking p38α in adipose tissues exhibited a lean phenotype and improved metabolism. To our surprise, adipocyte-specific deletion of p38α using the aP2-recombinase (Cre) line caused minimal effects on the morphology of interscapular brown adipose tissue (iBAT), the UCP-1 expression in iBAT, mitochondrial function, and body temperature (BT), as well as oxygen consumption and carbon dioxide production in adult mice. Interestingly, we found that genetic ablation of p38α in adipose tissues not only facilitated WAT browning upon cold stress but also prevented diet-induced obesity. The effect of adipocyte-specific p38α deficiency on WAT browning was subsequently verified by using the Adipoq-Cre line. Consistently, pharmaceutical inhibition of p38α promoted the browning of WAT and had beneficial effects. Further study revealed that p38α deficiency promoted white-to-beige adipocyte reprogramming in a cell-autonomous and cell type–specific manner. Mechanistically, suppression of p38α in WAT could stimulate the UCP-1 transcription through the PKA/CREB pathway. Our study indicates that p38α in WAT represents an exciting pharmacological target to combat obesity and metabolic diseases.

## Results

### Mice lacking p38α in adipose tissues display a lean phenotype and improved metabolism

**Fig 1:**
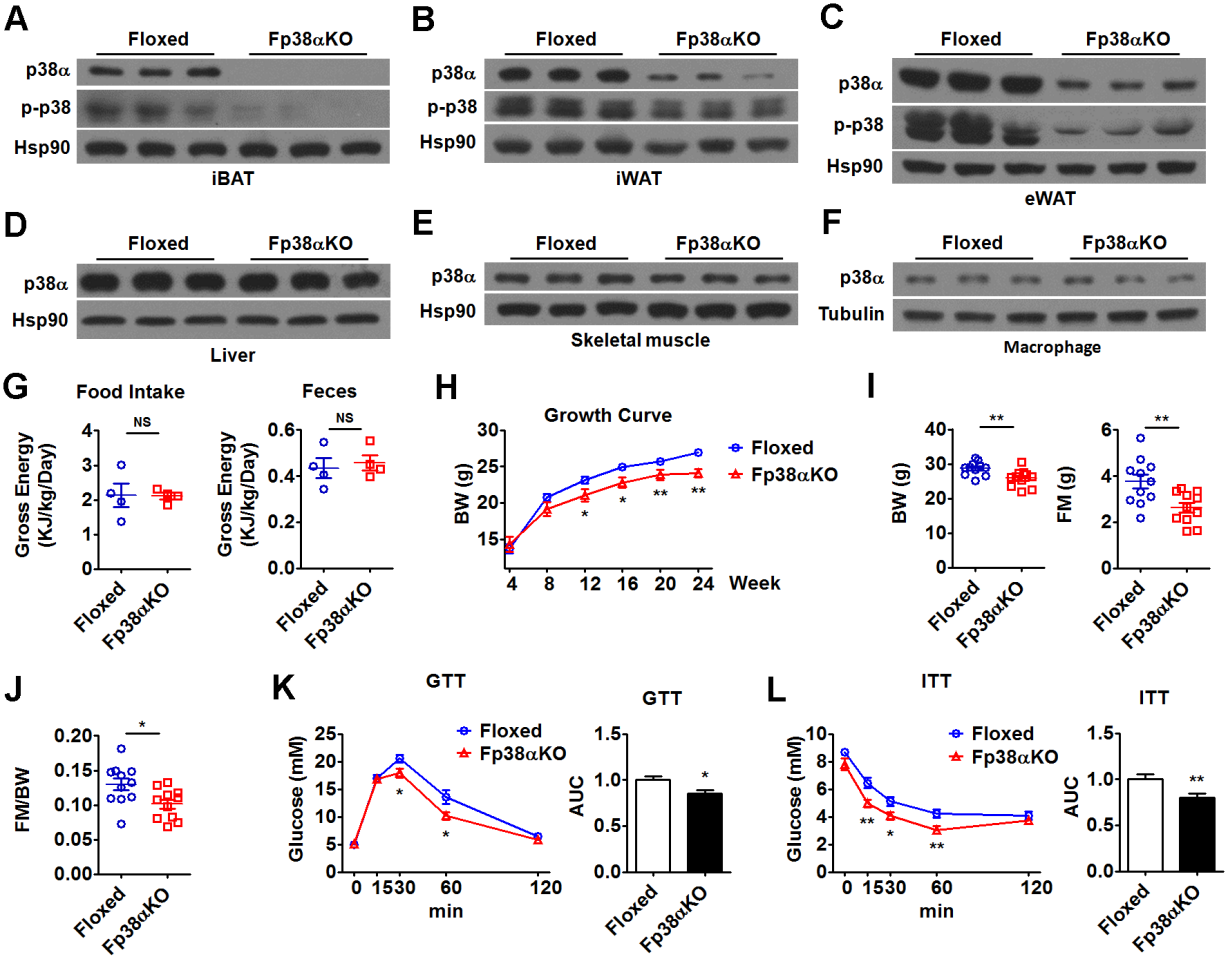
Adipocyte-specific deletion of p38α leads to a lean phenotype and increased glucose tolerance and insulin sensitivity. (A-C) Representative western blots of p38α and p-p38 in iBAT (A), iWAT (B), and eWAT (C) from Floxed and Fp38αKO mice as indicated. (D-F) Representative western blots of p38α in other tissues, including liver (D), skeletal muscle (E), and macrophages (F) from Floxed and Fp38αKO mice as indicated. (G) Cumulative gross energy intake and feces of Floxed and Fp38αKO mice for 24 h (*n* = 4 per group). Mice were maintained at RT. See also S1 Data. (H) Growth curve of Floxed (*n* = 15) and Fp38αKO (*n* = 8–13) mice maintained at RT. See also S1 Data. (I and J) BW, FM, and FM to BW ratio (FM/BW) of Floxed and Fp38αKO mice maintained at RT (*n* = 11 per group). See also S1 Data. (K and L) GTT (K, *n* = 8 per group) and ITT (L, *n* = 8 per group) in Floxed and Fp38αKO mice. AUCs were calculated. See also S1 Data. Means ± SEM are shown. *p < 0.05; **p < 0.01. AUC, area under curve; BW, body weight; eWAT, epididymal white adipose tissue; FM, fat mass; GTT, glucose tolerance test; iBAT, interscapular brown adipose tissue; ITT, insulin tolearance test; iWAT, inguinal white adipose tissue; NS, not significant; RT, room temperature.

To investigate the role of adipocyte p38α in vivo, we generated adipocyte-specific p38α knockout (Fp38αKO) mice using the Cre-lox system (p38α^f/f^; aP2-Cre^+/–^). As controls, floxed p38α (Floxed) mice that did not express Cre recombinase were used. As expected, p38α protein expression was greatly reduced in the iBAT, inguinal white adipose tissue (iWAT), and epididymal white adipose tissue (eWAT) of Fp38αKO mice as compared to Floxed mice (Fig 1A–1C, Fig A-C in S1 Fig, S1 Data). Accordingly, the protein levels of p-p38 were markedly decreased in iBAT, iWAT, and eWAT of Fp38αKO mice, suggesting that the p38 signaling in adipose tissues was greatly impaired (Fig 1A–1C, Fig A-C in S1 Fig, S1 Data). Also as expected, the protein levels of p38α were not changed in the liver and skeletal muscle of Fp38αKO mice as compared to Floxed mice (Fig 1D and 1E, Fig D and E in S1 Fig, S1 Data). We also determined the p38α protein levels in the macrophages to see whether aP2 Cre-mediated deletion of p38α could be detected in this cell type. Consistent with previous studies [27–29], we did not observe any decrease in p38α expression in intraperitoneal macrophages derived from the Fp38αKO mice (Fig 1F, Fig F in S1 Fig, S1 Data). Accordingly, the amount of either macrophages or neutrophils was not different in the peripheral blood between Floxed and Fp38αKO mice (Fig G in S1 Fig, S1 Data).

Fp38αKO mice were fertile and displayed normal energy intake and excretion when maintained at RT (Fig 1G, S1 Data). Growth curve analysis for body weight (BW) revealed that adult Fp38αKO mice had reduced BW compared to age-matched Floxed mice at RT (Fig 1H and 1I, S1 Data). The results of body composition analysis suggest that the lean phenotype of Fp38αKO mice might be due to the reduction in fat mass (FM), since lean mass (LM) was not affected (Fig 1I and 1J, Fig H in S1 Fig, S1 Data). The hematoxylin-eosin staining (HE staining) of iWAT and eWAT revealed that the size of adipocytes was smaller in Fp38αKO mice compared to Floxed mice at RT (Fig I-L in S1 Fig, S1 Data). Consistent with the lean phenotype, both glucose tolerance and insulin sensitivity were increased in Fp38αKO mice (Fig 1K and 1L, S1 Data). Similar results were obtained in a glucose tolerance test (GTT) when the glucose dose was adjusted on the basis of LM (Fig M in S1 Fig, S1 Data) [30]. Moreover, the levels of glucose and TGs in Fp38αKO mice were lower than those in Floxed mice at RT (Fig N in S1 Fig, S1 Data).

### Deletion of p38α in adipose tissues facilitates WAT browning upon cold stress

**Fig 2:**
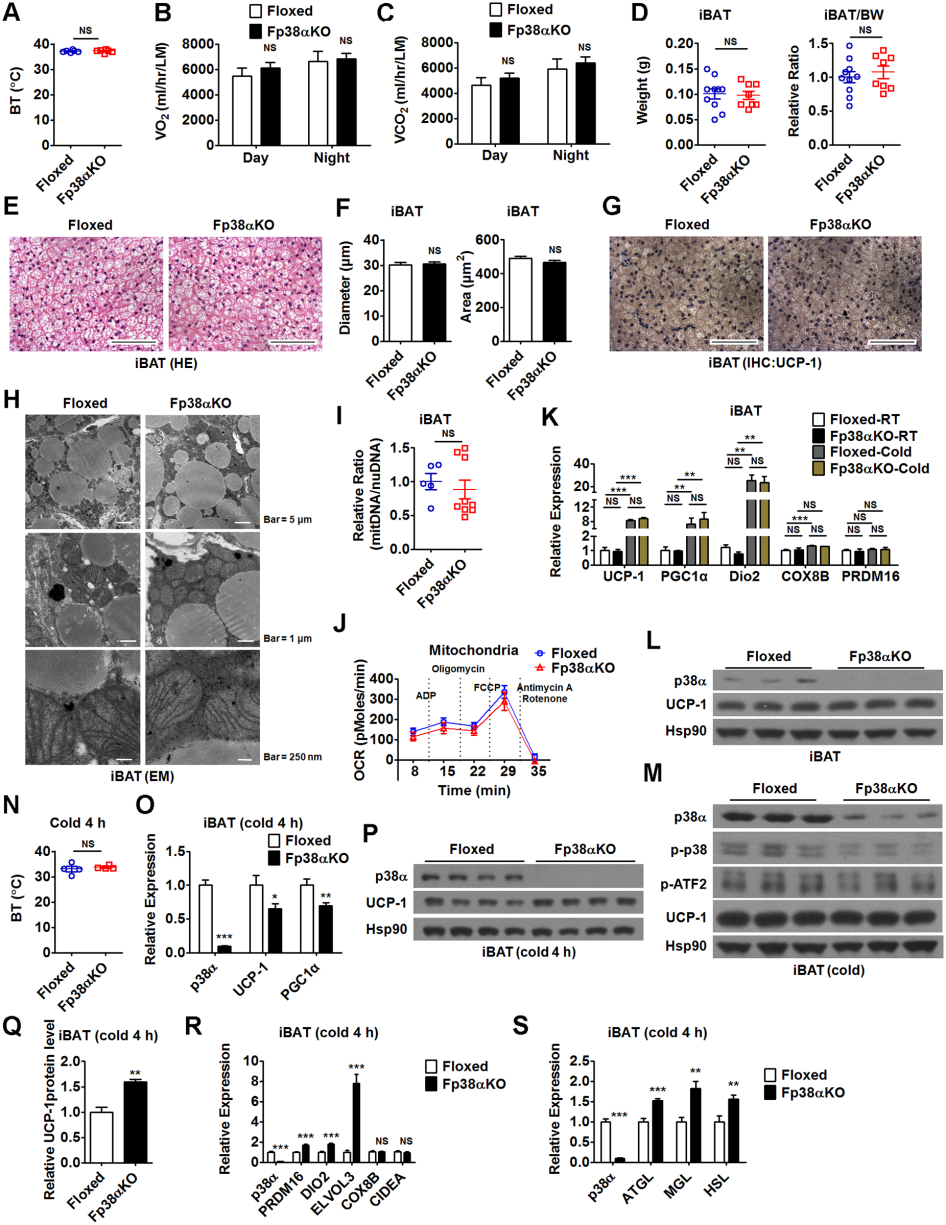
Loss of p38α in adipose tissues causes minimal effects on BAT. (A) BT of Floxed and Fp38αKO mice maintained at RT (*n* = 6 per group). See also S1 Data. (B and C) VO_2_ (B) and VCO_2_ (C) in Floxed and Fp38αKO mice maintained at RT (*n* = 4 per group). The values were normalized by LM. See also S1 Data. (D) iBAT weight and relative iBAT weight to BW ratio (iBAT/BW) of Floxed (*n* = 10) and Fp38αKO (*n* = 8). See also S1 Data. (E-G) Representative HE staining of iBAT (E), diameter and cross-sectional area of adipocytes in iBAT (F), and representative UCP-1 staining of iBAT (G) from Floxed and Fp38αKO mice maintained at RT. Bars: 100 μm. See also S1 Data. (H) Representative EM images of iBAT from Floxed and Fp38αKO mice maintained at RT at low (top), medium (middle), and high (bottom) magnification, as indicated. (I) Relative mitDNA to nuDNA ratio in unilateral iBAT of Floxed (*n* = 5) and Fp38αKO (*n* = 9) mice maintained at RT. See also S1 Data. (J) OCR of ADP, Oligomycin, FCCP, and Antimycin A/Rotenone-treated mitochondria derived from iBAT of Floxed and Fp38αKO mice exposed to cold for 2 d (*n* = 4 per group). See also S1 Data. (K) Relative mRNA levels of UCP-1, PGC1α, DIO2, COX8B, and PRDM16 in iBAT from Floxed and Fp38αKO mice maintained at RT (*n* = 6 per group) or exposed to cold for 2 d (*n* = 8 per group). See also S1 Data. (L and M) Representative western blots of UCP-1 in iBAT from Floxed and Fp38αKO mice maintained at RT (L) or exposed to cold for 2 d (M). (N) BT of Fp38αKO and Floxed mice exposed to cold for 4 h (*n* = 4 per group). See also S1 Data. (O) Relative mRNA levels of UCP-1 and PGC1α in iBAT from Floxed (*n* = 7–8) and Fp38αKO (*n* = 8) mice exposed to cold for 4 h. See also S1 Data. (P and Q) Representative western blots (P) and densitometry analysis (Q) of UCP-1 in iBAT from Floxed and Fp38αKO mice exposed to cold for 4 h. The densities of UCP-1 bands were quantitated and normalized to Hsp90 (*n* = 4 per group). See also S1 Data. (R and S) Relative mRNA levels of PRDM16, DIO2, ELVOL3, COX8B, and CIDEA(R), ATGL, MGL, and HSL (S) in iBAT from Floxed (*n* = 7–8) and Fp38αKO (*n* = 6–8) mice exposed to cold for 4 h. See also S1 Data. Means ± SEM are shown. *p < 0.05; **p < 0.01; ***p < 0.001. ADP, adenosine diphosphate; ATGL, adipose triglyceride lipase; BAT, brown adipose tissue; BT, body temperature; BW, body weight; CIDEA, cell death-inducing DNA fragmentation factor, alpha subunit-like effector A; COX8B, cytochrome c oxidase subunit 8B; DIO2, deiodinase 2; ELVOL3, elongation of very long chain fatty acids (FEN1/Elo2, SUR4/Elo3, yeast)-like 3; EM, electron microscopy; FCCP, carbonyl cyanide 4-(trifluoromethoxy)phenylhydrazone; HE staining, hematoxylin-eosin staining; HSL, hormone-sensitive lipase; iBAT, interscapular brown adipose tissue; IHC, immunohistochemistry; LM, lean mass; MGL, monoglyceride lipase; mitDNA, mitochondrial DNA; NS, not significant; nuDNA, nuclear DNA; OCR, oxygen consumption rate; PGC1α, peroxisome proliferative activated receptor gamma coactivator 1α; PRDM16, positive regulatory domain containing 16; RT, room temperature; UCP-1, uncoupling protein 1; VCO_2_, carbon dioxide production; VO_2_, oxygen consumption.

Since p38α has been shown to act as a central regulator of cAMP/PKA signaling and controls the transcription of UCP-1 in brown adipocytes [17,25], we speculated that the Fp38αKO mice would have reduced BT and/or altered energy expenditure. To our surprise, we did not observe any significant changes in BT, oxygen consumption, or carbon dioxide production in Fp38αKO mice compared to Floxed mice maintained at RT (Fig 2A–2C, S1 Data). To test whether loss of p38α in adipose tissue would affect cold-induced adaptive thermogenesis, we exposed Fp38αKO mice to a cold environment for 2 d. The change of BW after 2 d of cold exposure was not different between Floxed and Fp38αKO mice (Fig A in S2 Fig, S1 Data). However, the difference in BT between Fp38αKO and Floxed mice still could not be detected after cold challenge for 2 d (Fig B in S2 Fig, S1 Data), suggesting that p38α deficiency in adipose tissues could not impair the adaptations to cold exposure.

Consistently, the weight of iBAT, the histological morphology of iBAT, and the size of adipocytes in iBAT from Fp38αKO mice appeared indistinguishable from Floxed mice either at RT or after 2 d of cold exposure (Fig 2D–2F, Fig C-E in S2 Fig, S1 Data). The difference in iBAT morphology and the size of adipocytes in iBAT still could not be detected between Floxed and Fp38αKO mice after 7 d of cold exposure (Fig F and G in S2 Fig, S1 Data). The staining results of UCP-1 in iBAT were similar in Floxed and Fp38αKO mice maintained at RT (Fig 2G). Electron microscopy images of iBAT of Fp38αKO mice at RT revealed that the mitochondrial content and morphology were not altered (Fig 2H). The ratio of mitochondrial DNA (mitDNA) to nuclear DNA (nuDNA) was not different in iBAT between Floxed and Fp38αKO mice either maintained at RT or after 2 d of cold exposure (Fig 2I, Fig H in S2 Fig, S1 Data). The oxygen consumption rate (OCR) of the isolated iBAT mitochondria was comparable between Floxed and Fp38αKO mice after 2 d of cold exposure (Fig 2J, S1 Data). Consistently, the mRNA levels of those genes related to mitochondria function were either not or only slightly altered in the iBAT of Fp38αKO mice at RT or after 2 d of cold exposure, compared to Floxed mice (Fig I and J in S2 Fig, S1 Data). These results suggest that the mitochondrial function was not affected in the iBAT of Fp38αKO mice.

In agreement with the above findings, the mRNA levels of UCP-1 and other thermogenic genes were not changed in the iBAT of Fp38αKO mice compared to Floxed mice either at RT or after 2 d of cold exposure (Fig 2K, S1 Data). Additionally, the mRNA expression of those genes involved in fatty acid metabolism was either not or only slightly altered in the iBAT of Fp38αKO mice compared to Floxed mice after 2 d of cold exposure (Fig K in S2 Fig, S1 Data). Accordingly, the protein levels of UCP-1 were also not changed in the iBAT of Fp38αKO mice compared to Floxed mice either at RT or after 2 d of cold exposure (Fig 2L and 2M). Interestingly, the protein levels of p-ATF2 were slightly decreased in the iBAT of Fp38αKO mice compared to Floxed mice, but the differences did not quite reach statistical significance (Fig 2M, Fig L in S2 Fig, S1 Data). Since quantitative real-time PCR results revealed that p38α and p38β are the most abundant isoforms, and the expression of p38δ is very low in mouse iBAT (Fig M in S2 Fig, S1 Data), we determined the protein levels of p38β and p38γ in the iBAT of Fp38αKO mice. We found that the protein abundance of both p38β and p38γ was similar in iBAT between Floxed and Fp38αKO mice (Fig N and O in S2 Fig). We also measured the protein levels of tyrosine hydroxylase (TH), a marker of sympathetic innervations in iBAT of Fp38αKO mice, and found that the TH protein levels were not changed in iBAT from Fp38αKO mice compared to Floxed mice either at RT or in a cold environment for 2 d, indicating that sympathetic outflow was comparable between Floxed and Fp38αKO mice (Fig P in S2 Fig).

To examine whether Fp38αKO mice were unable to maintain BT upon acute cold exposure, mice were exposed to cold for 4 h. However, the difference in BT between Fp38αKO and Floxed mice still could not be detected after 4 h of cold challenge (Fig 2N, S1 Data). Interestingly, after an acute cold challenge for 4 h, a significant decrease in mRNA levels of UCP-1 and peroxisome proliferative activated receptor gamma coactivator 1α (PGC1α) was observed in iBAT from Fp38αKO mice compared to Floxed mice (Fig 2O, S1 Data), indicating that there is a defect in iBAT of Fp38αKO mice. Although the mRNA expression of UCP-1 was decreased in the iBAT of Fp38αKO mice, the UCP-1 protein levels were compensatorily increased in the iBAT of Fp38αKO mice after the acute cold exposure, compared to Floxed mice (Fig 2P and 2Q, S1 Data). We also analyzed the mRNA expression of thermogenic genes and genes involved in fatty acid metabolism in the iBAT of these mice. We found that the mRNA levels of positive regulatory domain containing 16 (PRDM16), deiodinase 2 (DIO2), and elongation of very long chain fatty acids (FEN1/Elo2, SUR4/Elo3, yeast)-like 3 (ELVOL3)—as well as adipose triglyceride lipase (ATGL), monoglyceride lipase (MGL), and hormone-sensitive lipase (HSL)—were all elevated in the iBAT of Fp38αKO mice after the acute cold exposure compared to Floxed mice, suggesting that the transcription of other thormogenic genes and lipolysis-related genes was compensatorily increased during acute cold exposure (Fig 2R and 2S, Fig Q in S2 Fig, S1 Data). In addition, the nonesterified fatty acid (NEFA) levels were also comparable between Floxed and Fp38αKO mice either maintained at RT or after acute cold exposure (Fig R in S2 Fig, S1 Data). There were no differences in the creatine kinase activity in serum, gastrocnemius (GAS) muscle, and heart between Floxed and Fp38αKO mice (Fig S in S2 Fig, S1 Data). These results suggest that the supply of fatty acids in Fp38αKO mice was normal.

**Fig 3:**
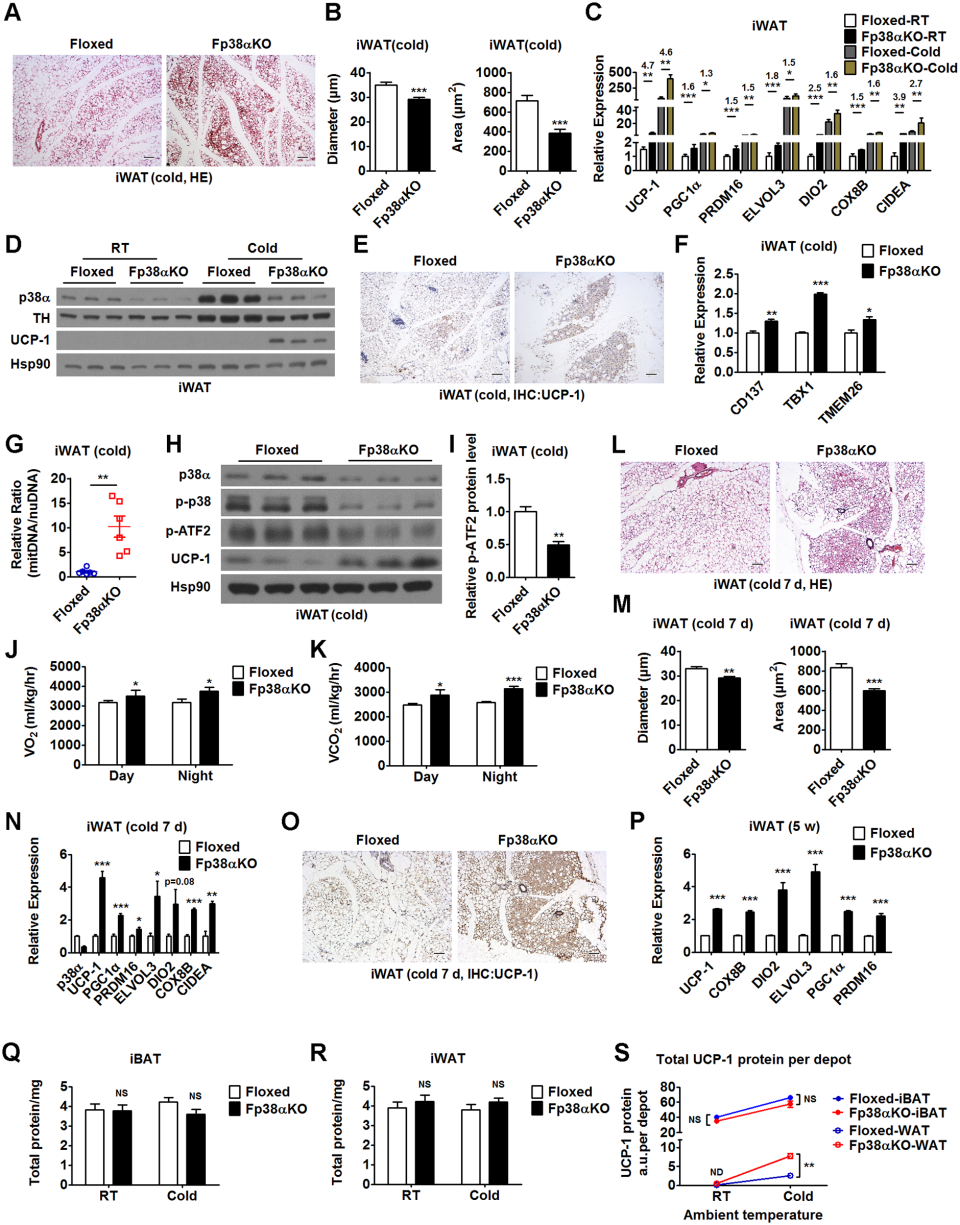
Ablation of p38α in adipose tissues facilitates the browning of WAT. (A and B) Representative HE staining of iWAT (A), diameter and cross-sectional area of adipocytes in iWAT (B) from Floxed and Fp38αKO mice exposed to cold for 2 d. Bars: 100 μm. See also S1 Data. (C) Relative mRNA levels of UCP-1, PGC1α, ELOVL3, DIO2, COX8B, and CIDEAin iWAT from Floxed and Fp38αKO mice maintained at RT or exposed to cold for 2 d (RT, *n* = 4 per group; Cold, *n* = 8–16 per group). Fold of change was indicated (Floxed versus Fp38αKO). See also S1 Data. (D) Representative western blots of UCP-1 and TH in iWAT from Floxed and Fp38αKO mice maintained at RT or exposed to cold for 2 d. (E) Representative UCP-1 staining of iWAT from Floxed and Fp38αKO mice exposed to cold for 2 d. Bars: 100 μm. (F) Relative mRNA levels of CD137, TBX1, and TMEM26 in iWAT from Floxed (*n* = 3) and Fp38αKO (*n* = 6) mice exposed to cold for 2 d. See also S1 Data. (G) Relative mitDNA to nuDNA ratio in unilateral iWAT of Floxed and Fp38αKO mice exposed to cold for 2 d (*n* = 6 per group). See also S1 Data. (H) Representative western blots of p38α, p-p38, p-ATF2, and UCP-1 in iWAT from Floxed and Fp38αKO mice exposed to cold for 2 d. (I) Relative p-ATF2 protein levels in iWAT of Floxed and Fp38αKO mice exposed to cold for 2 d. The densities of p-ATF2 bands were quantitated and normalized to Hsp90 (*n* = 3 per group). See also S1 Data. (J and K) VO_2_ (J) and VCO_2_ (K) within 48 h after CL316,243 injection in Floxed (*n* = 5) and Fp38αKO (*n* = 4) mice maintained at RT. See also S1 Data. (L and M) Representative HE staining of iWAT (L), diameter and cross-sectional area of adipocytes in iWAT (M) from Floxed and Fp38αKO mice exposed to cold for 7 d. Bars: 100 μm. See also S1 Data. (N) Relative mRNA levels of UCP-1, PGC1α, PRDM16, ELOVL3, DIO2, COX8B, and CIDEA in iWAT from Floxed and Fp38αKO mice exposed to cold for 7 d (*n* = 4 per group). See also S1 Data. (O) Representative UCP-1 staining of iWAT from Floxed and Fp38αKO mice exposed to cold for 7 d. Bars: 100 μm. (P) Relative mRNA levels of UCP-1, COX8B, DIO2, ELOVL3, PGC1α, and PRDM16 in iWAT from 5-wk-old Floxed (*n* = 6) and Fp38αKO (*n* = 6) mice maintained at RT. See also S1 Data. (Q–S) Total amounts of protein in iBAT (*n* = 6–9) and iWAT (*n* = 5–11), and total UCP-1 protein per depot (*n* = 3) in both iBAT and iWAT of Floxed and Fp38αKO mice maintained at RT or exposed to cold for 2 d. See also S1 Data. Means ± SEM are shown. *p < 0.05; **p < 0.01; ***p < 0.001. ATF2, activating transcription factor 2; ATGL, adipose triglyceride lipase; BAT, brown adipose tissue; BT, body temperature; BW, body weight; CIDEA, cell death-inducing DNA fragmentation factor, alpha subunit-like effector A; COX8B, cytochrome c oxidase subunit 8B; DIO2, deiodinase 2; ELVOL3, elongation of very long chain fatty acids (FEN1/Elo2, SUR4/Elo3, yeast)-like 3; HE staining, hematoxylin-eosin staining; HSL, hormone-sensitive lipase; iBAT, interscapular brown adipose tissue; IHC, immunohistochemistry; iWAT, inguinal white adipose tissue; MGL, monoglyceride lipase; mitDNA, mitochondrial DNA; ND, not detectable; NS, not significant; nuDNA, nuclear DNA; OCR, oxygen consumption rate; PGC1α, peroxisome proliferative activated receptor gamma coactivator 1α; PRDM16, positive regulatory domain containing 16; RT, room temperature; TBX1, T-box 1; TH, tyrosine hydroxylase; TMEM26, transmembrane protein 26; UCP-1, uncoupling protein 1; VCO_2_, carbon dioxide production; VO_2_, oxygen consumption; WAT, white adipose tissue.

Since increased browning of WAT has been observed in many knockout mouse models that show improved metabolism, the finding of improved metabolism in Fp38αKO mice prompted us to investigate the browning of WAT in these mice without apparent alterations in BAT function. Interestingly, profoundly increased browning was observed in iWAT from Fp38αKO mice exposed to a cold environment for 2 d, as indicated by significantly increased emergence of multilocular adipocytes, reduced adipocyte size, increased vascular density, increased expression of UCP-1 and other thermogenic or beige adipocyte genes, and increased ratio of mitDNA to nuDNA (Fig 3A–3G, S1 Data). In contrast to iBAT, iWAT showed significantly decreased p-ATF2 levels in Fp38αKO mice compared to Floxed mice after 2 d of cold exposure (Fig 3H and 3I, S1 Data). The levels of p-CREB (Ser133) were increased in the iWAT of Fp38αKO mice after 2 d of cold exposure, which might contribute to the up-regulation of UCP-1 in these mice (Fig A in S3 Fig). Since it is widely assumed that the browning of WAT can increase energy expenditure, we speculated that the energy expenditure would be affected in Fp38αKO mice after the induction of WAT browning. As expected, increased energy expenditure was observed within 48 h after β3-adrenoceptor agonist (CL316,243) injection in Fp38αKO mice compared to Floxed mice (Fig 3J and 3K, Fig B in S3 Fig, S1 Data). Similar results were obtained in mice exposed to a cold environment for 7 d before analysis (cold-adapted mice) (Fig C in S3 Fig, S1 Data). These results further indicate that deletion of p38α in adipose tissues facilitates WAT browning upon cold stress.

Increased emergence of multilocular adipocytes, reduced adipocyte size, and increased expression of UCP-1 and other thermogenic genes were also observed in the iWAT from Fp38αKO mice exposed to cold for 7 d compared to Floxed mice (Fig 3L–3O, S1 Data), further suggesting that lacking p38α in adipose tissues could lead to an increase in WAT browning upon cold stress. In addition, distinct histological morphology and smaller adipocyte size; increased expression of thermogenic genes, including UCP-1; and increased ratio of mitDNA to nuDNA were observed in the iWAT from 5-wk-old Fp38αKO mice maintained at RT, which was indicative of enhanced browning (Fig 3P, Fig D-F in S3 Fig, S1 Data). Accordingly, smaller adipocyte size was also observed in eWAT from 5-wk-old Fp38αKO mice compared to age-matched Floxed mice (Fig G-H in S3 Fig, S1 Data).

To test whether the increased browning of iWAT observed in adult Fp38αKO mice upon cold exposure and 5-wk-old Fp38αKO mice maintained at RT was due to an increase in sympathetic input, we measured the protein levels of TH in iWAT of these mice. We found that the protein levels of TH in iWAT were not altered in adult Fp38αKO mice compared to Floxed mice upon cold stress for 2 d (Fig 3D). Similarly, we did not detect any changes in TH protein levels in iWAT from 5-wk-old Fp38αKO mice maintained at RT compared to age-matched Floxed mice (Fig I in S3 Fig). These results indicate that the increased browning of iWAT observed in cold-exposed adult Fp38αKO mice or 5-wk-old Fp38αKO mice maintained at RT was independent of sympathetic action.

It has been reported that different adipocyte-specific Cre lines displayed different degrees of efficiency and specificity. Another Cre line driven by the mouse adiponectin promoter regions within the brown adipocyte cell line (BAC) transgene (Adipoq-Cre) was bred to p38α^f/f^ mice to generate Ap38αKO mice. As expected, p38α protein expression was greatly reduced in iBAT and iWAT of Ap38αKO mice (Fig J and K in S3 Fig). Consistent with the observation in Fp38αKO mice, the UCP-1 levels were elevated in iWAT of cold-exposed Ap38αKO mice (Fig K in S3 Fig), further supporting the notion that ablation of p38α in adipose tissues could promote WAT browning in the adaptive response to cold environments.

To see whether the adipogenesis was affected in the iWAT of Fp38αKO mice after cold exposure, in vivo BrdU-labeling experiments were performed. We found that the proportion of BrdU-positive adipocytes in iWAT was not different between Floxed and Fp38αKO mice after cold challenge for 7 d (Fig L and M in S3 Fig, S1 Data), suggesting that the adipogenesis was not affected in the iWAT of Fp38αKO mice. Additionally, we determined the mRNA levels of p38 isoforms in mouse iWAT and found that p38α and p38β are the most abundant isoforms in mouse iWAT, and the expression of p38δ is very low in mouse iWAT (Fig N in S3 Fig, S1 Data). We then examined the protein levels of p38β and p38γ in the iWAT of Fp38αKO mice and found that the protein expression of both p38β and p38γ was not altered in Fp38αKO mice either maintained at RT or after 2 d of cold exposure compared to Floxed mice (Fig O and P in S3 Fig).

According to a previous study [31], the total protein of adipose tissues would increase after cold exposure for 7 wk, especially for iBAT. To take this into consideration, we quantitatively determined the total protein content in iBAT and iWAT, and UCP-1 content per mg protein, then calculated the total UCP-1 content per depot in both genotypes. We found that total protein content in iBAT and iWAT was comparable between Floxed and Fp38αKO mice either maintained at RT or exposed to cold for 2 d (Fig 3Q and 3R, S1 Data). The total UCP-1 content per depot for iBAT was similar in both Floxed and Fp38αKO mice (Fig 3S, S1 Data). In contrast, increased total UCP-1 per depot for iWAT was observed in Fp38αKO mice compared to Floxed mice after 2 d of cold exposure (Fig 3S, S1 Data).

### Ablation of p38α in adipose tissues prevents diet-induced obesity

**Fig 4:**
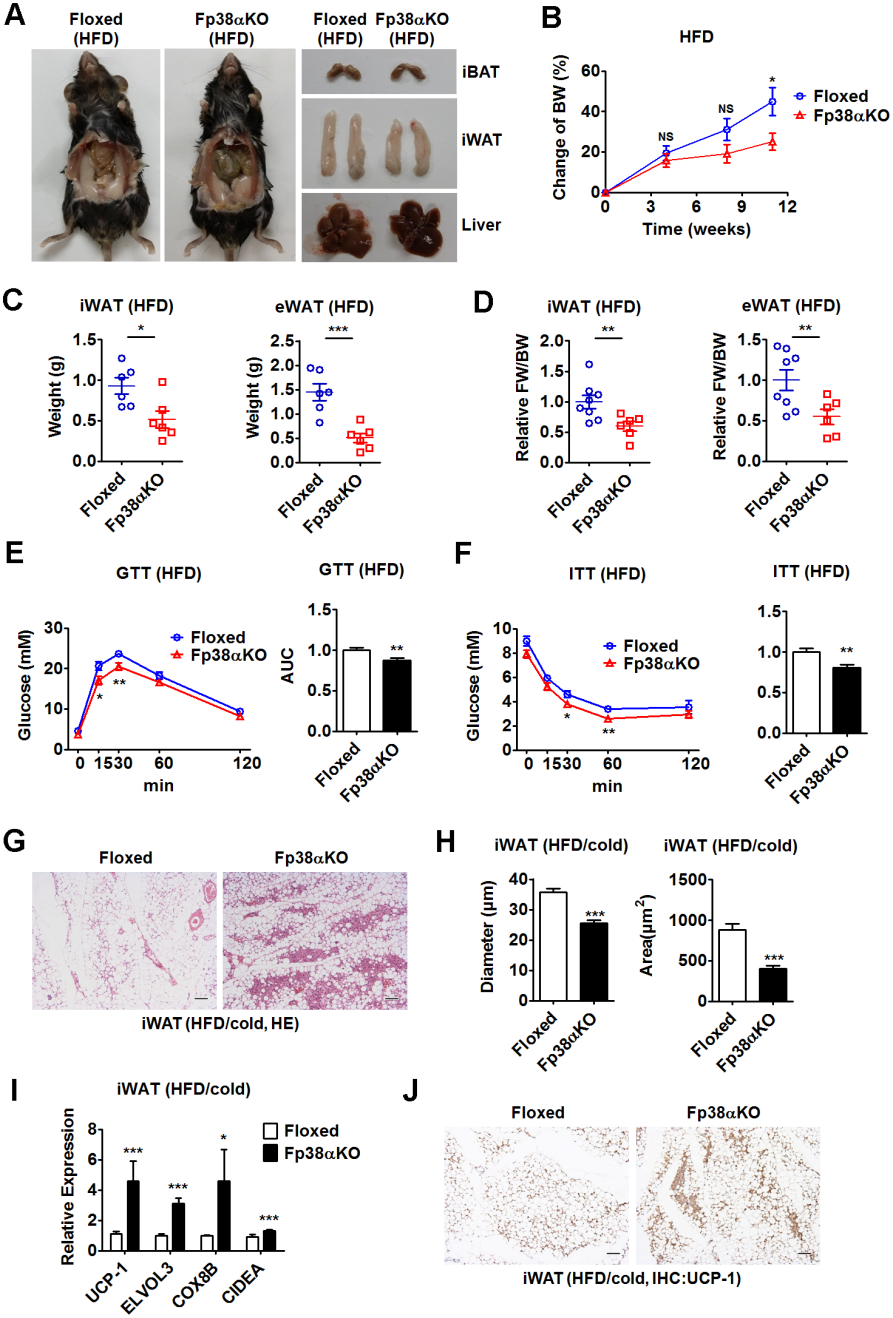
Deletion of p38α in adipose tissues prevents diet-induced obesity and improves metabolism. (A) Representative photographs of Floxed and Fp38αKO mice after HFD feeding and iBAT, iWAT, and liver dissected from these mice. (B) Change of BW of Floxed and Fp38αKO mice upon HFD feeding (*n* = 6 per group). See also S1 Data. (C and D) Weight of iWAT and eWAT (C), relative FW of iWAT and eWAT (D) to BW ratio of Floxed (*n* = 6–8 per group) and Fp38αKO (*n* = 6 per group) mice after HFD feeding. See also S1 Data. (E and F) GTT (E) and ITT (F) in Floxed and Fp38αKO mice after HFD feeding (*n* = 8 per group). AUCs were calculated. See also S1 Data. (G and H) Representative HE staining of iWAT (G), diameter and cross-sectional area of adipocytes in iWAT (H) from HFD-fed Floxed and Fp38αKO mice exposed to cold for 2 d. Bars: 100 μm. See also S1 Data. (I) Relative mRNA levels of UCP-1, ELOVL3, COX8B, and CIDEA in iWAT from HFD-fed Floxed (*n* = 6) and Fp38αKO (*n* = 5–6) mice exposed to cold for 2 d. See also S1 Data. (J) Representative UCP-1 staining of iWAT from HFD-fed Floxed and Fp38αKO mice exposed to cold for 2 d. Bars: 100 μm. Means ± SEM are shown. *p < 0.05; **p < 0.01; ***p < 0.001. AUC, area under curve; BW, body weight; CIDEA, cell death-inducing DNA fragmentation factor, alpha subunit-like effector A; COX8B, cytochrome c oxidase subunit 8B; ELVOL3, elongation of very long chain fatty acids (FEN1/Elo2, SUR4/Elo3, yeast)-like 3; eWAT, epididymal white adipose tissue; FW, fat weight; GTT, glucose tolerance test; HE staining, hematoxylin-eosin staining; HFD, high-fat diet; iBAT, interscapular brown adipose tissue; ITT, insulin tolerance test; iWAT, inguinal white adipose tissue; NS, not significant; UCP-1, uncoupling protein 1.

To determine the effect of adipocyte-specific p38α deficiency on diet-induced obesity, we treated adult Fp38αKO and Floxed mice with a high-fat diet (HFD). After 3 months of HFD challenge, adult Fp38αKO mice gained less BW and had smaller iWAT and eWAT weight compared to Floxed mice (Fig 4A–4C, S1 Data). No significant difference was observed in iBAT weight and GAS muscle weight between these 2 groups of mice after HFD treatment (Fig A and B in S4 Fig, S1 Data). When normalized to the BW, the relative weight of either iWAT or eWAT was significantly lower in Fp38αKO mice than that in Floxed mice (Fig 4D, S1 Data). Although the liver weight of HFD-fed adult Fp38αKO mice was similar to that of Floxed mice (Fig C in S4 Fig, S1 Data), histological analysis and Oil Red O staining results revealed that the fatty liver was improved in HFD-fed adult Fp38αKO mice (Fig D and E in S4 Fig). In agreement with these findings, the GTT and insulin tolerance test (ITT) experiments revealed that HFD-fed adult Fp38αKO mice displayed improved glucose tolerance and insulin sensitivity (Fig 4E and 4F, S1 Data). As expected, we observed increased browning as indicated by formation of multilocular adipocytes, reduced adipocyte size, and increased expression of UCP-1 and other thermogenic genes in iWAT from HFD-fed Fp38αKO mice after 2 d of cold exposure (Fig 4G–4J, S1 Data). The adipocyte size was also reduced in eWAT from HFD-fed Fp38αKO mice exposed to cold for 2 d (Fig F and G in S4 Fig, S1 Data). Together, these results suggest that ablation of p38α in adipose tissues was able to prevent diet-induced obesity and improve metabolism, which might be attributed to the increased browning potential of WAT.

Although we could not detect any differences in either oxygen consumption or carbon dioxide production between HFD-fed Floxed and Fp38αKO mice maintained at RT (Fig H and I in S4 Fig, S1 Data), we did see an increase in macrophage infiltration in the iWAT of Fp38αKO mice after 2 d of cold exposure, as evident from increased number of CD68^+^ cells (Fig J and K in S4 Fig, S1 Data). We also found that the mRNA expression of M2-related genes was increased, while the mRNA levels of proinflammatory cytokines were reduced in the iWAT of these HFD-fed Fp38αKO mice after cold exposure (Fig L and M in S4 Fig, S1 Data). These findings are in agreement with our current knowledge of the browning process. In an in vivo BrdU-labeling experiment, we found that the proportion of BrdU^+^ adipocytes in iWAT from HFD-fed Fp38αKO mice was reduced compared to Floxed mice after cold challenge for 7 d (Fig N and O in S4 Fig, S1 Data). Interestingly, the percentage of BrdU^−^UCP-1^+^ adipocytes relative to the total numbers of UCP-1^+^ adipocytes was higher in the iWAT of these HFD-fed Fp38αKO mice (80%) than that in control animals (40%), suggesting increased conversion or transdifferentiation of existing white adipocytes (UCP-1^−^) into beige adipocytes (UCP-1^+^) in these HFD-fed Fp38αKO mice during cold exposure (Fig O in S4 Fig, S1 Data).

### Pharmaceutical inhibition of p38α promotes browning of WAT and improves metabolism

**Fig 5:**
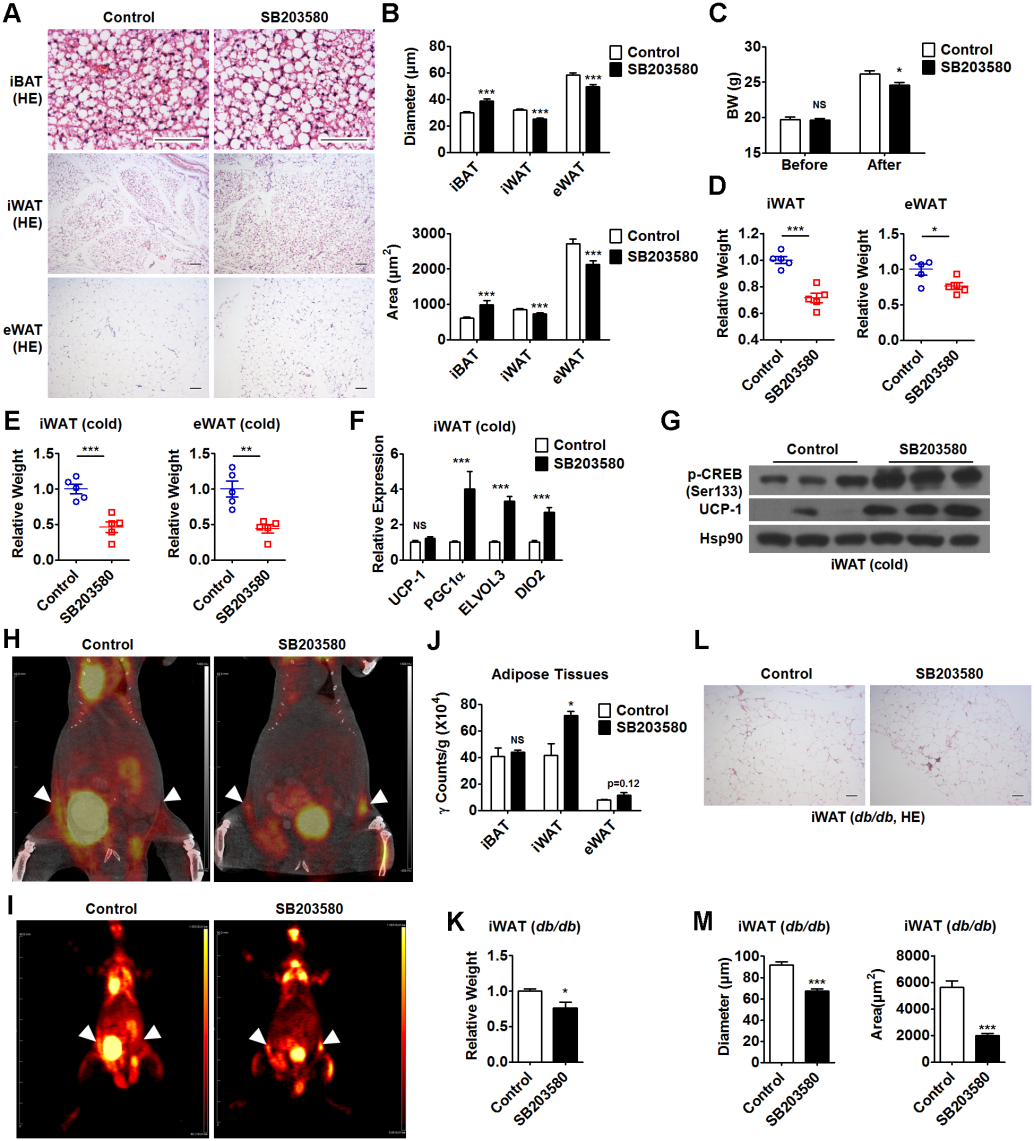
Pharmaceutical inhibition of p38α reduces adiposity and enhances the browning of WAT. (A and B) Representative HE staining of iBAT, iWAT, and eWAT (A), diameter and cross-sectional area (B) of adipocytes in these adipose tissues from SB203580-treated C57BL/6J mice at 2 d postinjection. Bars: 100 μm. See also S1 Data. (C) BW of C57BL/6J mice before and after 4 wk of SB203580 treatment (control: *n* = 4, SB203580: *n* = 5). See also S1 Data. (D and E) Relative weight of iWAT and eWAT from C57BL/6J mice after 4 wk of SB203580 treatment. These mice were maintained at RT (D, *n* = 5 per group) or exposed to cold for 2 d before analysis (E, *n* = 5 per group) as indicated. See also S1 Data. (F) Relative mRNA levels of UCP-1, PGC1α, ELOVL3, and DIO2 in iWAT from C57BL/6J mice after 4 wk of SB203580 treatment (*n* = 10 per group). These mice were exposed to cold for 2 d before analysis. See also S1 Data. (G) Representative western blots of p-CREB (Ser133) and UCP-1 in iWAT from C57BL/6J mice after 4 wk of SB203580 treatment. These mice were exposed to cold for 2 d before analysis. (H and I) Representative PET/CT images (H) and PET images (I) of C57BL/6J mice after 4 wk of SB203580 treatment. These mice received a daily CL316,243 injection for 8 d before ^18^F-FDG injection. White dashed triangles represent the anatomical sites of iWAT. (J) Ex vivo measured ^18^F-FDG uptake in iBAT, iWAT, and eWAT to tissue weight ratio by γ counter (*n* = 3 per group). See also S1 Data. (K) Relative weight of iWAT from *db/db* mice after 3 wk of SB203580 treatment (*n* = 5 per group). See also S1 Data. (L and M) Representative HE staining of iWAT (L), diameter and cross-sectional area of adipocytes in iWAT (M) from *db/db* mice after 3 wk of SB203580 treatment. Bars: 100 μm. See also S1 Data. Means ± SEM are shown. *p < 0.05; **p < 0.01; ***p < 0.001. BW, body weight; CREB, cAMP-response element binding protein; CT, computed tomography; DIO2, deiodinase 2; ELVOL3, elongation of very long chain fatty acids (FEN1/Elo2, SUR4/Elo3, yeast)-like 3; eWAT, epididymal white adipose tissue; HE staining, hematoxylin-eosin staining; iBAT, interscapular brown adipose tissue; iWAT, inguinal white adipose tissue; NS, not significant; PET, positron emission tomography; PGC1α, peroxisome proliferative activated receptor gamma coactivator 1α; RT, room temperature; UCP-1, uncoupling protein 1; WAT, white adipose tissue.

The finding that Fp38αKO mice were resistant to diet-induced obesity encouraged us to test whether pharmaceutically targeting p38α using SB203580 could have a similar beneficial effect. We found that 48 h of SB203580 treatment reduced the adipocyte size in iWAT and eWAT from C57BL/6J mice, accompanied by an increase of adipocyte size in iBAT from the same animal (Fig 5A and 5B, S1 Data). We also found that 4 wk of SB203580 treatment led to a lean phenotype, as evident from a decrease in BW and relative weight of iWAT, eWAT, and iBAT, but had no effect on liver weight (Fig 5C and 5D, Fig A and B in S5 Fig, S1 Data). Additionally, glucose levels were decreased after 4 wk of SB203580 treatment (Fig C in S5 Fig, S1 Data). The decrease in iWAT and eWAT weight, as wells as in glucose levels, became more evident in SB203580-treated mice upon cold exposure for 2 d (Fig 5E, Fig D in S5 Fig, S1 Data), which was accompanied by increased expression of UCP-1 and other thermogenic genes in iWAT (Fig 5F and 5G, S1 Data), suggesting the increased capacity of browning in these mice might contribute to the decreased adiposity and glucose levels. To be noted, the protein levels of p-CREB (Ser133) were also increased in SB203580-treated mice upon cold exposure, which might contribute to the elevation of UCP-1 expression (Fig 5G).

The positron emission tomography (PET) tracer ^18^F-FDG was used to monitor the CL316,243-induced brown adipocyte recruitment into WAT in C57BL/6J mice after 4 wk of SB203580 treatment. As expected, PET/computed tomography (CT) showed an increase in ^18^F-FDG uptake in iWAT of SB203580-treated mice compared to control mice (Fig 5H and 5I). Ex vivo measurement of ^18^F-FDG uptake in different adipose tissues revealed that the ^18^F-FDG uptake in iWAT was increased in mice receiving SB203580 treatment (Fig 5J, S1 Data). A small increase in ^18^F-FDG uptake in eWAT of SB203580-treated mice was also observed, although the difference did not quite reach statistical significance (Fig 5J, S1 Data). We did not observe any differences in ^18^F-FDG uptake in iBAT between SB203580-treated mice and control mice. The ^18^F-FDG uptake in skeletal muscle was decreased in SB203580-treated mice, although there was no evidence for myofiber-type conversion (Fig E and F in S5 Fig, S1 Data). These results further suggest that SB203580 treatment could promote WAT browning without affecting BAT function in mice.

SB203580 was also employed to treat obese *db/db* mice. Obese *db/db* mice gained less weight and had lower BW after receiving 3 wk of SB203580 treatment compared to control animals (Fig G and H in S5 Fig, S1 Data). Further investigation revealed that 3 wk of SB203580 treatment not only reduced the weight of iWAT but also decreased the adipocyte size in iWAT from these *db/db* mice (Fig 5K–5M, S1 Data). As expected, we observed increased infiltration of CD68^+^ macrophages in the iWAT from SB203580-treated *db/db* mice, accompanied by increased mRNA expression of M2-related genes and decreased mRNA expression of proinflammatory cytokines (Fig I-L in S5 Fig, S1 Data). Together, these results indicate that pharmaceutically targeting p38α might have a beneficial effect on metabolism.

### p38α deficiency promotes white-to-beige adipocyte reprogramming

**Fig 6:**
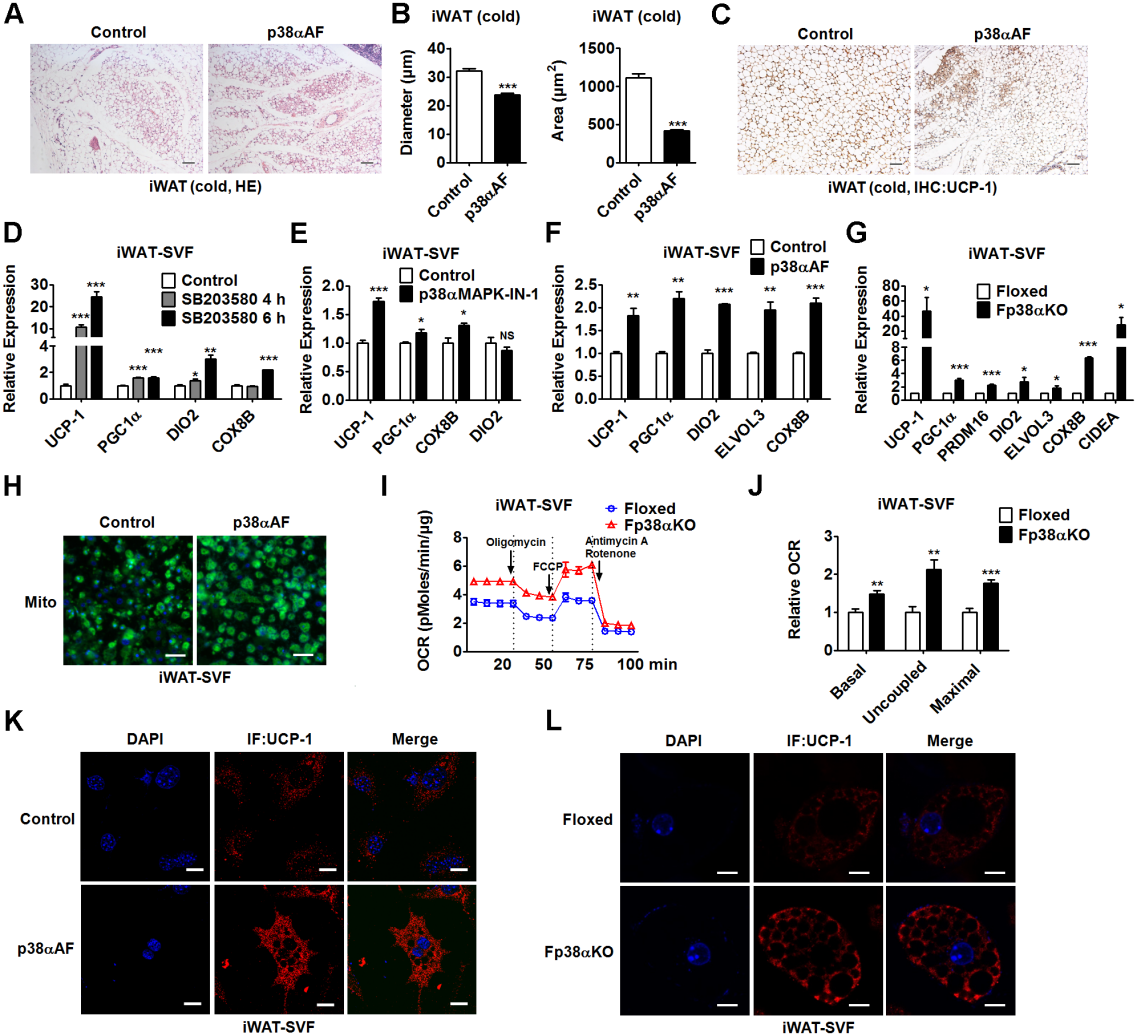
The effect of p38α inhibition or deficiency on WAT browning is cell autonomous. (A-C) Representative HE staining of iWAT (A), diameter and cross-sectional area of adipocytes in iWAT (B), and representative UCP-1 staining of iWAT (C) from C57BL/6J mice after injection of Ad-p38αAF into the fat pad of iWAT. These mice were exposed to cold for 2 d before analysis. Bars: 100 μm. See also S1 Data. (D-F) Relative mRNA levels of UCP-1, PGC1α, DIO2, COX8B, and/or ELVOL3 in iWAT-SVF-derived matured adipocytes treated with SB203580 for 4 or 8 h (D, *n* = 4 per group), or p38α-specific inhibitor (p38αMAPK-IN-1) for 4 h (E, *n* = 4 per group), or infected with Lenti-p38αAF (F, *n* = 3 per group). See also S1 Data. (G) Relative mRNA levels of UCP-1, PGC1α, PRDM16, DIO2, ELVOL3, COX8B, and CIDEA in matured adipocytes derived from iWAT-SVF of Floxed and Fp38αKO mice (*n* = 4 per group). See also S1 Data. (H) Representative fluorescence staining of Mito in matured iWAT-SVF-derived adipocytes infected with Lenti-p38αAF. Bars: 25 μm. (I and J) OCR of Oligomycin, FCCP, and Antimycin A/Rotenone-treated matured adipocytes derived from iWAT-SVF of Floxed and Fp38αKO mice (I) and the AUC of OCR (J) as indicated (*n* = 5 per group). See also S1 Data. (K and L) Representative UCP-1 staining of iWAT-SVF-derived matured adipocytes after infection with Lenti-p38αAF (K) or matured adipocytes derived from iWAT-SVF of Floxed and Fp38αKO mice (L). Bars: 10 μm. Means ± SEM are shown. *p < 0.05; **p < 0.01; ***p < 0.001. Ad-p38αAF, adenovirus expressing p38αAF; AUC, area under the curve; CIDEA, cell death-inducing DNA fragmentation factor, alpha subunit-like effector A; COX8B, cytochrome c oxidase subunit 8B; DIO2, deiodinase 2; ELVOL3, elongation of very long chain fatty acids (FEN1/Elo2, SUR4/Elo3, yeast)-like 3; FCCP, carbonyl cyanide 4-(trifluoromethoxy)phenylhydrazone; HE staining, hematoxylin-eosin staining; IHC, immunohistochemistry; iWAT, inguinal white adipose tissue; Lenti-p38αAF, lentivirus expressing p38αAF; MAPK, mitogen-activated protein kinase; Mito, mitochondria; NS, not significant; OCR, oxygen consumption rate; PGC1α, peroxisome proliferative activated receptor gamma coactivator 1α; PRDM16, positive regulatory domain containing 16; SVF, stromal vascular fraction; UCP-1, uncoupling protein 1; WAT, white adipose tissue.

Given that genetic ablation of p38α was capable of increasing browning of WAT without affecting sympathetic activation, we hypothesized that p38α in WAT might function in a cell-autonomous manner. To test our hypothesis, we injected adenovirus-expressing p38αAF (Ad-p38αAF), which is a dominant-negative form of p38α, into the iWAT of C57BL/6J mice (Fig A and B in S6 Fig, S1 Data). As expected, a distinct histological morphology, significantly reduced adipocyte size, and increased staining of UCP-1 were observed in Ad-p38αAF-infected mice exposed to cold for 2 d (Fig 6A–6C, S1 Data). Additionally, Ad-p38αAF-infected mice had decreased glucose and TG levels compared to control mice (Fig C in S6 Fig, S1 Data).

To further explore the role of p38α in WAT, we performed cell-based experiments. Interestingly, we found that SB203580 treatment increased the mRNA levels of UCP-1 and DIO2 in matured 3T3L1 adipocytes while suppressing the mRNA expression of UCP-1 and other thermogenic genes in matured brown adipocytes differentiated from either a BAC or stromal vascular fraction (SVF) isolated from iBAT (iBAT-SVF) of C57BL/6J mice (S6D–S6F Fig, S1 Data). Consistently, the mRNA expression of UCP-1 and other thermogenic genes was up-regulated by SB203580 treatment in a time-dependent manner in matured adipocytes derived from SVF isolated from iWAT (iWAT-SVF) of C57BL/6J mice (Fig 6D, S1 Data). Given that p38α is the major p38 isoform in either matured iBAT-SVF-derived adipocytes or matured iWAT-SVF-derived adipocytes (S6G Fig, S1 Data), similar results were obtained by using a p38α inhibitor (p38αMAPK-IN-1) (Fig 6E, Fig H in S6 Fig, S1 Data).

Additionally, the mRNA expression of UCP-1 and other thermogenic genes was elevated in matured 3T3L1 adipocytes infected with lentivirus expressing p38αAF (Lenti-p38αAF) (Fig I and J in S6 Fig, S1 Data). We did not detect any effects of Lenti-p38αAF on lipid accumulation in these matured 3T3L1 adipocytes (Fig K in S6 Fig). Similarly, infection of Lenti-p38αAF increased the mRNA levels of UCP-1 and other thermogenic genes in iWAT-SVF-derived matured adipocytes (Fig 6F, Fig L in S6 Fig, S1 Data). To substantiate the evidence that inhibition of p38 signaling has differential effects on thermogenic program in brown adipocytes and white adipocytes, matured adipocytes derived from either iWAT-SVF or iBAT-SVF of Fp38αKO mice were used. We found that loss of p38α did not influence the adipogenic capacity of iWAT-SVF-derived cells (S6M and S6N Fig). In agreement with the results obtained by using either inhibitors or Lenti-p38αAF, the mRNA expression of thermogenic genes was increased in matured adipocytes derived from iWAT-SVF of Fp38αKO mice (Fig 6G, S1 Data). In contrast to what we observed in iWAT-SVF-derived matured adipocytes, the mRNA levels of thermogenic genes were decreased in matured adipocytes derived from iBAT-SVF of Fp38αKO mice (S6O Fig, S1 Data).

An increase in mitochondria staining was also observed in iWAT-SVF-derived matured adipocytes infected with Lenti-p38αAF, indicating that the thermogenic program was activated upon p38α inhibition (Fig 6H). Accordingly, by using Agilent Seahorse XF24 Analyzer, we found that basal, uncoupled, and maximal OCRs were all significantly increased in matured adipocytes derived from iWAT-SVF of Fp38αKO mice as compared to Floxed mice, further suggesting that loss of p38α could enhance mitochondrial function in white adipocytes (Fig 6I and 6J, S1 Data). Consistently, the immunofluorescence staining of UCP-1 in iWAT-SVF-derived matured adipocytes was increased by Lenti-p38αAF infection (Fig 6K). Consistent with above findings using Lenti-p38αAF, we found that the UCP-1 expression was significantly increased in matured adipocytes derived from iWAT-SVF of Fp38αKO mice compared to control cells (Fig 6L). In addition, an increase in UCP-1 and DIO2 mRNA expression was also observed in matured adipocytes derived from WAT-SVF of Ap38αKO mice compared to control cells (Fig P in S6 Fig, S1 Data). Together, these results suggest that inhibition of p38α might promote white-to-beige adipocyte reprogramming in a cell-autonomous manner.

### Suppression of p38α activates thermogenic program through PKA/CREB pathway in WAT

**Fig 7:**
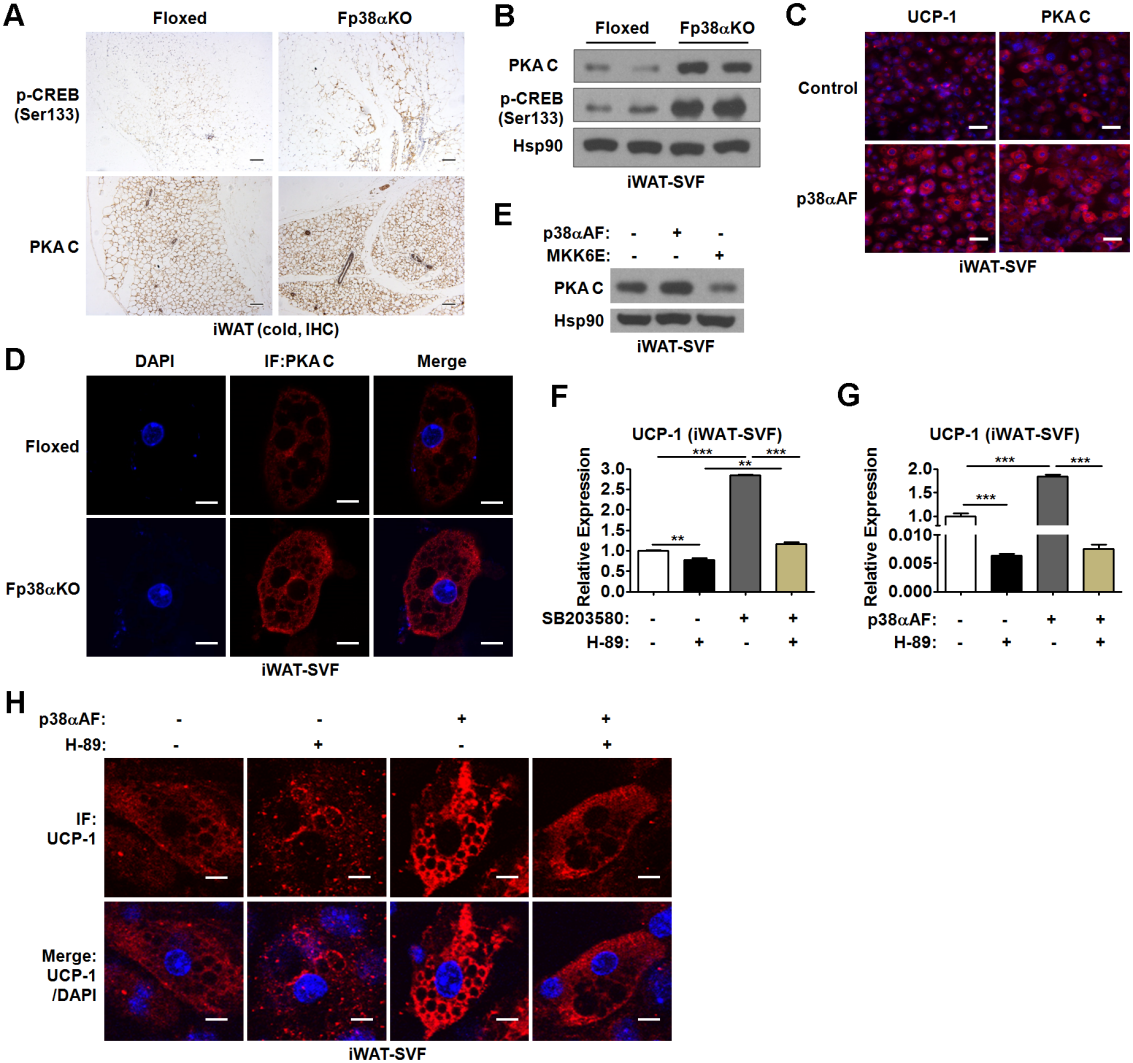
PKA/CREB pathway mediates the effect of p38α deficiency in WAT. (A) Representative p-CREB (Ser133) and PKA C staining of iWAT from Floxed and Fp38αKO mice exposed to cold for 2 d. Bars: 100 μm. (B) Representative western blots of PKA C and p-CREB (Ser133) in matured adipocytes derived from iWAT-SVF of Floxed and Fp38αKO mice. (C) Representative fluorescence staining of UCP-1 and PKA C in matured iWAT-SVF-derived adipocytes infected with Lenti-p38αAF. Bars: 25 μm. (D) Representative immunofluorescence staining of PKA C in matured adipocytes derived from iWAT-SVF of Floxed and Fp38αKO mice. Bars: 10 μm. (E) Representative western blots of PKA C in matured iWAT-SVF-derived adipocytes infected with Lenti-p38αAF or Lenti-MKK6E as indicated. (F) Relative mRNA levels of UCP-1 in matured iWAT-SVF-derived adipocytes treated with SB203580 and/or H-89 as indicated (*n* = 3 per group). See also S1 Data. (G) Relative mRNA levels of UCP-1 in matured iWAT-SVF-derived adipocytes infected with Lenti-p38αAF and/or treated with H-89 as indicated (*n* = 3 per group). See also S1 Data. (H) Representative immunofluorescence staining of UCP-1 in matured iWAT-SVF-derived adipocytes infected with Lenti-p38αAF and/or treated with H-89 as indicated. Bars: 10 μm. Means ± SEM are shown. **p < 0.01; ***p < 0.001. CREB, cAMP-response element binding protein; IF, immunofluorescence; IHC, immunohistochemistry; iWAT, inguinal white adipose tissue; Lenti-MKK6E, lenntivirus expressing a constitutive active mutant of a mitogen-activated protein kinase kinase; Lenti-p38αAF, lentivirus expressing p38αAF; PKA, protein kinase A; PKA C, PKA catalytic subunit; SVF, stromal vascular fraction; UCP-1, uncoupled protein 1; WAT, white adipose tissue.

In agreement with the findings that the protein levels of p-CREB (Ser133) were elevated in iWAT from either Fp38αKO mice (Fig 7A, Fig A in S3 Fig) or SB203580-treated mice (Fig 5G) after 2 d of cold exposure, we also observed that the protein levels of p-CREB (Ser133) were increased in either iWAT-SVF-derived matured adipocytes lacking p38α (Fig A in S7 Fig) or iWAT-SVF-derived matured adipocytes infected with Lenti-p38αAF (Fig B in S7 Fig). Consistent with these results, chromatin immunoprecipitation (ChIP) assay revealed that loss of p38α resulted in an enrichment of p-CREB (Ser133) on 2 cAMP response elements (CRE2 and CRE4) in the UCP-1 enhancer in iWAT-SVF-derived matured adipocytes (Fig C and D in S7 Fig). These results prompted us to explore the upstream signaling mediator that might contribute to the increased p-CREB (Ser133) levels.

Since it has been reported that PKA is able to phosphorylate CREB at Ser 133, we tested whether PKA expression was altered when p38α was absent. Interestingly, increased staining of PKA catalytic subunit (PKA C) was observed in iWAT from cold-exposed adult Fp38αKO mice, accompanied by increased staining of p-CREB (Ser133) in the same animal (Fig 7A). Similar results were obtained in iWAT from 5-wk-old Fp38αKO mice maintained at RT (Fig E in S7 Fig). Consistently, the protein levels of PKA C and p-CREB (Ser133) were elevated in matured adipocytes derived from iWAT-SVF of Fp38αKO mice (Fig 7B). In agreement with these findings, either inhibition of p38α by Lenti-p38αAF or genetic deletion of p38α increased the immunofluorescence staining of PKA C in iWAT-SVF-derived matured adipocytes (Fig 7C and 7D). Additionally, the levels of phosphorylated PKA substrates in matured 3T3L1 adipocytes infected with Lenti-p38αAF were elevated, suggesting the PKA activity was increased by p38α inhibition (Fig F in S7 Fig). To test whether activation of p38 signaling could suppress the expression of PKA C, a lentivirus expressing a constitutive active mutant of an MAPK kinase (Lenti-MKK6E) was employed. As expected, in contrast to the effect of Lenti-p38αAF on the protein levels of PKA C, Lenti-MKK6E infection led to a decrease in PKA C protein levels in iWAT-SVF-derived matured adipocytes (Fig 7E). These data suggest that p38α in WAT is a negative regulator of PKA/CREB pathway.

To test whether the PKA/CREB pathway mediated the effect of p38α inhibition on the thermogenic program in iWAT-SVF-derived matured adipocytes, H-89, an inhibitor of PKA, was employed. We found that suppression of PKA activity by H-89 was able to attenuate the effect of p38α inhibition either by SB203580 or Lenti-p38αAF on the UCP-1 mRNA expression in iWAT-SVF-derived matured adipocytes (Fig 7F and 7G, S1 Data). Consistently, we found that H-89 could abolish the effect of Lenti-p38αAF on the protein levels of UCP-1 in iWAT-SVF-derived matured adipocytes by using immunofluorescence staining (Fig 7H). Together, our results suggest that inhibition of p38α might stimulate the thermogenic program through PKA/CREB pathway in WAT.

Interestingly, we found that the protein levels of p-CREB (Ser133) were reduced instead of increased in SB203580-treated BAC, matured adipocytes derived from iBAT-SVF of Fp38αKO mice, and iBAT from Fp38αKO mice maintained at RT compared to their controls, respectively (Fig G-I in S7 Fig). In contrast to p-CREB, we found that the protein levels of p-ATF2 were not only decreased in the matured adipocytes derived from the iWAT-SVF of Fp38αKO mice but also reduced in SB203580-treated matured adipocytes derived from iWAT-SVF of C57BL/6J mice (Fig J in S7 Fig). Thus, our data suggest that the molecular mechanism underlying the transcriptional regulation of UCP-1 in WAT is distinct from that in BAT.

**Fig 8:**
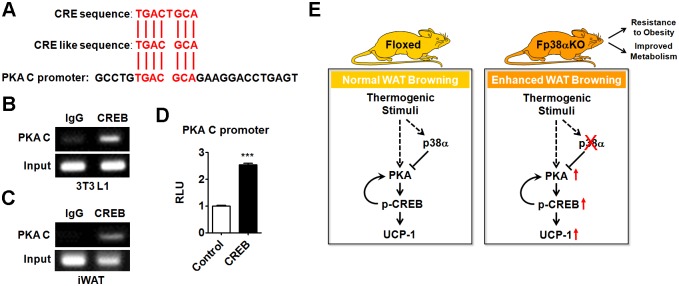
Transcriptional regulation of PKA C by CREB and schematic diagram of the working model. (A) Sequence alignment of classical CRE or CRE-like sequence with PKA C promoter. (B and C) Representative results of ChIP analysis of CREB enrichment on putative CRE in the PKA C promoter in 3T3L1 cells (B) and iWAT (C) of C57BL/6J mice respectively. (D) Luciferase reporter assay to analyze the PKA C promoter activity in CREB-transfected HEK293T cells (*n* = 6 per group). See also S1 Data. (E) Schematic diagram of the working model for the roles p38α in WAT browning. Means ± SEM are shown. ***p < 0.001. ChIP, chromatin immunoprecipitation; CRE, cAMP response element; CREB, cAMP-response element binding protein; IgG, immunoglobulin G; iWAT, inguinal white adipose tissue; PKA C, PKA catalytic subunit; RLU, relative light unit; UCP-1, uncoupling protein 1; WAT, white adipose tissue.

More interestingly, we found a putative CRE in the promoter region of the PKA C gene (Fig 8A). ChIP assay using the CREB antibody revealed an enrichment of CREB on this putative CRE in the PKA C promoter in either matured 3T3L1 adipocytes or iWAT of C57BL/6J mice (Fig 8B and 8C). Accordingly, luciferase assay demonstrated that overexpression of CREB could enhance the activity of PKA C promoter containing this putative CRE in HEK293T cells (Fig 8D, S1 Data). These results suggested that CREB could regulate the expression of PKA C at transcriptional level. In this mode, PKA and CREB form a positive feedback loop that serves to activate a thermogenic program that leads to white-to-beige phenotypic switching during adaption to cold (Fig 8E).

Based on our current data, we propose that adipose p38α is a critical regulator of energy homeostasis. p38α functions as a brake of the PKA signaling pathway in WAT, thereby conferring robust and precise controls on the adaptation of adipose tissues to cold exposure and the whole-body energy metabolism (Fig 8E). Inhibition of p38α in WAT promotes browning, which could serve as a new therapeutic approach to combat obesity and improve metabolic homeostasis (Fig 8E).

## Discussion

Maintenance of a proper BT is essential for survival in homeotherms. The sophisticated mechanisms of thermoregulation may participate in the control of energy homeostasis and the development of metabolic disorders [32,33]. Since both brown and beige adipocytes are present in adult humans and have remarkable capacity to dissipate stored energy, these two types of adipocytes hold great promise to treat obesity and metabolic diseases [34,35]. In this study, we demonstrate that p38α signaling controls the development and formation of beige adipocytes in WAT. We found that genetic ablation of p38α in adipose tissues facilitated WAT browning during cold exposure. Moreover, loss of p38α in adipose tissues led to a lean phenotype, improved metabolism, and resistance to diet-induced obesity. Given that pharmaceutical inhibition of p38α promoted the browning of WAT and had beneficial effects, we propose that p38α in WAT could serve as an exciting pharmacological target to combat obesity and metabolic diseases.

p38α is activated in response to a variety of extracellular stimuli and mediates signal transduction from the cell surface to the nucleus. p38 has been long proposed to positively regulate the thermogenic program in brown adipocytes as a downstream mediator of cAMP/PKA signaling [25]. Our finding that the ablation of p38α in adipose tissues of mice caused minimal effects on thermogenic function of iBAT, as indicated by undetectable changes in UCP-1 expression, BT, morphology, and mitochondrial function, as well as energy expenditure in adult mice (Fig 2A–2M, Fig A-K in S2 Fig, S1 Data). These unexpected findings yielded from our study suggest that the loss of p38α in brown adipocytes is not as deleterious as we previously thought.

On the other hand, to our knowledge, the role of p38 in the browning of WAT, especially the regulation of the thermogenic program in WAT, has never been extensively studied. Recently, PKA–apoptosis signal-regulating kinase 1 (ASK1)-p38 axis has been shown to contribute to the induction of brown adipocyte–specific gene expression in response to cAMP signaling [36]. Either CL316,243 treatment or enhancing the expression of ASK1 was able to induce UCP-1 expression, which was accompanied by an increase in p-p38 levels. However, the effect of p38 inhibition on CL316,243-induced ASK1 activation and UCP-1 expression was not investigated in that study. Therefore, the roles of adipocyte p38 in the regulation of thermogenic program remain unclear. Here, our data suggest that p38α acts as a negative regulator of browning in WAT (Fig 8E). Suppression of p38α, instead of activation of p38α, is able to stimulate the thermogenic program through enhancing PKA signaling in WAT. Thus, p38α differentially regulates PKA signaling and the thermogenic program upon cold exposure in BAT and WAT. These findings also indicate that p38α plays an important role in coordinating energy homeostasis by controlling the thermogenic program in an adipose depot–specific manner.

Recent studies have greatly expanded our knowledge of beige adipocytes. In contrast to classic brown adipocytes, beige adipocytes express relatively low levels of theromogenic genes under nonstimulated conditions, which are dramatically induced upon cold exposure [8]. Although it is possible that common regulatory mechanisms may operate, since the thermogenic program in BAT and the browning of WAT are both stimulated by cold stress, it remains to be established whether cell type–specific mechanisms exist in brown and beige adipocytes [37]. In brown adipocytes, the activation of p38 by cAMP/PKA signaling stimulates the transcription of UCP-1 through direct phosphorylation of ATF2, which binds to a well-characterized enhancer located 2.5 kb upstream of the UCP-1 gene [25]. Whether the cAMP/PKA/p38/ATF2 cascade also plays a similar role in WAT browning remains unclear. In our study, we did observe the effect of loss of p38α on the phosphorylation of ATF2 in WAT during browning; however, the reduced levels of p-ATF2 could not explain the elevation of UCP-1 expression, suggesting there is a distinct regulatory mechanism in WAT (Fig 3H and 3I, S1 Data). Interestingly, we found genetic or pharmaceutical inhibition of p38α was able to stimulate rather than suppress the transcription of UCP-1 through PKA/CREB cascade in WAT. These findings suggest that cell type-specific signaling cascades exist in BAT and WAT, which provide a means to fine-tune the expression of UCP-1 in all adipose tissues across the whole body.

It has been widely accepted that beige fat is metabolically important, especially during cold exposure and nutrient overload [38–40]. Notably, obesity resistance in mice appears to be mostly related to browning of WAT rather than to adaptive thermogenesis of classic BAT, suggesting that beige fat is a key contributor to metabolic health [41]. Previous studies have clearly demonstrated that stimulating the browning process improves metabolism and protects mice from diet-induced obesity, whereas ablation of beige adipocytes results in metabolic dysfunction [42,43]. It has also been proposed that beige fat has nonthermogenic functions and regulates energy metabolism through various mechanisms in response to nutrient stress. In light of the presence of beige adipocytes in adult humans, these findings have been generating considerable interest, as understanding the molecular mechanisms underlying the browning of WAT could lead to novel therapeutic strategies for treating obesity and metabolic disorders [8,44]. Adipocyte p38 has been investigated before by using commercially available human adipocytes, preadipocytes, SVF, or directly using adipocytes collected from human subjects. These studies suggest that the adipocyte p38 is involved in the regulation of the response to inflammatory stress, cardiac natriuretic peptide-induced thermogenic program, and cellular aging [35,45–48]. More importantly, it has been reported that phosphorylation p38 was increased either in type 2 diabetic adipocytes or omental fat from obese women [49,50]. In our study, we observed that p38α deficiency in adipose tissues increased the browning in WAT, which was accompanied by resistance to obesity and improvement of metabolism. Mechanistically, p38α deficiency could promote white-to-beige adipocyte reprogramming in a cell-autonomous manner. Our findings, together with those reported by others [49,50], suggest that inhibition of the overactivated p38 in WAT may be beneficial for obese or diabetic subjects. Although we could not achieve WAT-specific delivery, our data suggest that pharmaceutical inhibition of p38α by SB203580 treatment is able to reduce the fat weight and glucose levels (Fig 5, S5 Fig, S1 Data). However, whether the pharmaceutical inhibition of p38α would promote white-to-beige adipocyte reprogramming in human adipocytes is not known and requires further study.

In agreement with previous reports [31,51], our results indicate that although there was a dramatic increase in the protein levels of UCP-1 in the iWAT of mice upon cold exposure, at the system level the contribution from classic brown-fat UCP-1-mediated thermogenesis would still predominate (Fig 3S, S1 Data). Therefore, the changes in UCP-1 levels in the iWAT of Fp38αKO mice upon cold exposure might not explain the whole phenotype we observed in this study. UCP-1-independent and/or nonthermogenic mechanisms need to be investigated in future studies.

Taken together, we establish an important role for p38α in the browning of WAT and energy homeostasis. Based on our findings, we propose that p38α in WAT could serve as a novel druggable target to combat obesity and metabolic diseases.

## Materials and methods

### Ethics statement

All animal protocols were approved by the Animal Care Committee of Institute for Nutritional Sciences (INS), Shanghai Institutes for Biological Sciences (SIBS), and Chinese Academy of Sciences (CAS) (Approval number 2015-AN-12). All in vivo experiments described in this study were in accordance with institutional guidelines for the care and use of animals.

### Mice

Mice with a targeted deletion of p38α in adipose tissues were generated by crossing the p38αflox/flox (p38α^f/f^) mice with transgenic mice expressing Cre-recombinase under the control of the fatty acid binding protein 4 promoter (aP2-Cre) (Fp38αKO mice) or the adiponectin promoter (Adipoq-Cre) (Ap38αKO mice). Littermates expressing no Cre (Floxed mice) were used as a control group throughout the experiments. Mice were fed 60 kcal% HFD for 3 mo since 6 wk old or injected with SB203580 (20 mg kg^−1^, Medchemexpress Company) every 7 d for 1 mo. Mice were fed 60 kcal% HFD for 5 wk. Then, these HFD-fed mice were maintained in a cold environment (4 °C) and injected intraperitoneally with BrdU (200 mg kg^−1^, Sigma) twice a day for 7 d. The *db/db* mice were injected with SB203580 (20 mg kg^−1^) every 7 d for 3 wk from 6 wk old. Mice used in this study were aged between 2 to 4 mo if not specially pointed out. Male mice were used in the experiments. Cold treatment was conducted by sending mice to a cold room (4 °C) for 2 d or 7 d supplied with food and water. For acute cold challenge, mice were exposed to cold (4 °C) only with water.

Mice were randomly assigned to each group; however, blinding was not possible. Mice with similar age or from same litters had the priority of use. During the experiments, mice were monitored daily. Any mice with significantly abnormal signs of rapid weight loss, inability to eat or drink, clinical symptomatology, toxicity, or unresponsiveness would be recorded, and the data from these mice were excluded for statistical analysis. We estimated the sample size by using an online program from http://www.powerandsamplesize.com/Calculators/ for animal study. If the known value is 1, expected value is 1.2 (20% difference between groups), standard deviation is 0.1, and alpha is 0.05, the power of the test will reach 0.93 when sample size is 3, or the power will reach 0.99 when sample size is 5. If the expected value is 1.1–1.5 (smaller difference), 5–10 or even more samples will be used to have the power bigger than 0.9.

### Measurement of body composition/temperature, energy expenditure, and gross energy input and output

The food intake was evaluated by weighing out the grams of food every 12 h (7 AM–7 PM day, 7 PM–7 AM night). Minispec TD-NMR Analysers were used to evaluate living body composition. Rectal temperature was measured with a model BAT-12 thermometer (Physitemp Instruments). To measure energy expenditure, mice with or without β3 agonist (CL316, 243 Sigma) (1 mg kg^−1^ BW) injection were placed in metabolic cages (Columbus Instruments) to assess their O_2_ consumption and CO_2_ production. Gross energy content of food and feces in 24 h was determined using oxygen bomb calorimeter (IKA Oxygen Bomb Calorimeter C 6000).

### GTT and ITT

For GTT, mice were fasted for 14 h and injected with D-glucose (2 g kg^−1^ BW or 2.5 g kg^−1^ LM) (Sigma) intraperitoneally. For ITT, mice were fasted for 4 h and injected with recombinant human insulin (1 U kg^−1^, Roche) intraperitoneally.

### Blood glucose, plasma TG, NEFA, and creatine kinase activity measurements

We measured mouse blood glucose levels with whole blood from the tail vein using a glucose meter (Abbott). For TG measurement, plasma was collected through fresh whole blood centrifuged for 10 min after 30 min of standing. TG and NEFA levels were measured using a commercial ELISA kit (Labassay). The activity of creatine kinase in plasma, skeletal muscle, and hearts was measured using an ELISA kit (Abnova).

### Mouse PET/CT and radioactivity measurements

To activate browning process in WATs, mice were treated with β3 agonist (CL316, 243 Sigma) (1 mg kg^−1^ BW) daily by intraperitoneal injections for 8 d at RT. Mice PET-CT imaging was performed by Siemens Inveon PET-CT Multimodality System. In brief, mice were fasted overnight, lightly anesthetized using 3% isofluorane, and injected with approximately 150 μCi of ^18^F-FDG into the tail vein. After that, the animal was permitted to roam freely in the cage for 1 h to uptake ^18^F-FDG. Subsequently, the animal was placed onto the imaging bed under 2% isofluorane anesthesia for the duration of imaging. All the PET/CT experiments were operated under RT. Tissues of mice after PET/CT imaging were ex vivo measured with γ counter (SN-695 γ RIA Counter) and corrected with tissue weight, respectively.

### Morphological studies

Mouse tissues were fixed in 4% paraformaldehyde and embedded in paraffin. Sections were stained with hematoxylin and eosin or oil red according to standard protocols. Immunohistochemical staining of paraffin sections was carried out with 1:50 anti-UCP-1 (Abcam), 1:50 p-CREB (Ser133) (CST), and 1:50 PKA C (CST) and detected by Inverted microscope (Olympus). The cell sizes and areas of adipose tissues were measured by Image J. Immunofluorescence staining of paraffin sections for BrdU incorporation was carried out with primary antibodies 1:50 anti-UCP-1 (Abcam), 1:100 BrdU (Santa Cruz), or 1:200 CD68 (Bio-Rad, Formerly AbD Serotec) and secondary antibodies Alexa Fluor 594 conjugated goat antibody to rabbit IgG, Alexa Fluor 488 conjugated goat antibody to mouse IgG, or Alexa Fluor 594 conjugated goat antibody to rat IgG (Invitrogen, 1:1,000). The Elecron microscopic observations were conducted through scanning electron microscope (PHILIPS CM120). All the representative images were repeated in at least 3 independent experiments.

### Flow cytometry analyses

Macrophages and neutrophils were isolated from blood of Floxed and Fp38αKO mice. RBCs were lysed using ACK lysis buffer. A single-cell suspension was used for staining cell surface markers F4/80, Gr-1, and Mac-1 (eBioscience) following standard protocols, and data acquisition was performed using a FACSAria II cytometer (BD). Flow cytometric data were analyzed with FLOWJO software.

### RNA isolation and real-time PCR

Total RNA was extracted from cells or tissues using TRIzol reagent (Invitrogen) in accordance with the manufacturer's instructions. One microgram of RNA was transcribed to complementary DNA with the RT Reagent Kit (Takara). Real-time PCR was carried out on the 7900 System (ABI) using SYBER Green Supermix (Takara). Primers used in this study were provided in S1 Table. Data were normalized to 18S and analyzed using the ΔΔCT method. To quantify the expression of p38 isoforms, absolute quantification of p38α, p38β, p38γ, and p38δ was performed. Four pairs of primers were designed to amplify fragments from mice cDNA, which were used as templates for standard curves. The other 4 pairs of primers were designed to perform regular real-time PCR to get specific copy numbers according to standard curves respectively.

### Protein preparation and western blotting

Proteins of cells or tissues were extracted by RIPA buffer. All protein samples were subjected to 5 ng/μL and immunoblot assay with the indicated antibodies. Hsp90 (CST) or α-tubulin (Sigma) were used as internal controls. Detailed information on the antibodies used in this study is provided in S2 Table. The representative blotting bands were repeated at least 3 times. The gray intensity of blotting bands was evaluated through Image J.

### Cell culture and differentiation

3T3L1 preadipocyte cell line was cultured in DMEM supplemented with 10% NCS (Gbico) and 1% penicillin/streptomycin at 37 °C with 5% CO_2_. SVF-derived preadipocytes were isolated as described previously from iWAT. Firstly, inguinal pads from 5-wk-old mice were minced and digested with 2% collagenase type I in DMEM for 30 min at 37 °C, followed by quenching with complete medium. Cell suspensions were centrifuged washed and filtered through a 70 μm strainer (BD Biosciences) and were plated onto 10 cm dishes in DMEM supplemented with 10% FBS and 1% penicillin/streptomycin (Invitrogen). BAC cell line was immortalized from SVF-derived preadipocytes from iBAT of newborn C57BL/6J mice by SV40 retriovirus and cultured in DMEM supplemented with 10% FBS and 1% penicillin/streptomycin at 37 °C with 5% CO_2_. Adipocyte differentiation of confluent cells—including 3T3L1, BAC, and SVF-derived preadipocytes—was induced in growth medium with 5 μg/ml insulin, 0.5 mM IBMX (Sigma), 1 μM DEX (Sigma), 1 nM T3, and 5 μM Rosiglitazone (Sigma) for 48 h and replaced with growth medium supplemented with insulin, T3, and Rosiglitazone for 4 d. After that, matured adipocytes were infected with lentivirus GFP, p38αAF, or MKK6E for 48 h. Ten micromoles of SB203580 (Merck/millipore)—the inhibitor of p38α and p38β—20 μM p38α MAPK–IN–1 (MCE), or 20μM H-89 (Selleck)—the inhibitor of PKA—were preincubated before other treatments or sample collection. Ten nanomoles of Forskolin (Sigma) were added to matured adipocytes for 3 h before RNA/immunofluorescence collection or 30 min before protein collection unless pointed out. HEK293T cell line was cultured in DMEM supplemented with 10% FBS and 1% penicillin/streptomycin at 37 °C with 5% CO_2_.

### Immunofluorescence

The mitochondria were stained by Mito-Tracker Green (Beyotime) for 30 min in DMEM. Cells were fixed with 4% paraformaldehyde and incubated with 1:50 anti-UCP-1 (Abcam) or 1:100 anti-PAK C (CST) or 1:100 anti-Perilipin (CST) antibody and further detected by secondary antibodies Alexa Fluor 594 conjugated goat antibody to rabbit IgG (Invitrogen 1:1,000). Cells were then washed in PBS and stained with 40, DAPI. Images were acquired by fluorescence microscopy (Zeiss System) or High Content Screening Microplate Imaging Reader (Thermo Fisher Scientific). The representative images were repeated in at least 3 independent experiments.

### Measurement of mitochondria number and mitochondrial respiration

Genome DNA from adipose tissues of Floxed and Fp38αKO mice was extracted using a DNA Mini Preparation Kit with Spin Column (Beyotime, Shanghai). The presence of amplifiable mitDNA and nuDNA in the extract was assayed through real-time PCR.

Differentiated primary adipocytes were trypsinized on day 8 after differentiation and plated into the XF24 V7 cell culture microplate. After 48 h, the OCRs were determined by a Seahorse Bioscience XF24 Extracellular Flux Analyzer (Seahorse Bioscience), with Oligomycin 2 μM, FCCP 1.5 μM and Antimycin A/Rotenone 1 μM injected during fixed time intervals. The OCRs were normalized by proteins in each well.

Mitochondrial respiration of BAT after cold exposure was determined using an XF24 Extracellular Flux Analyzer (Seahorse Bioscience) using 5 μg mitochondrial protein in a buffer containing 50 mM KCl, 4 mM KH2PO4, 5 mM HEPES, and 1 mM EGTA, 4% BSA, 10 mM Pyruvate, 5 mM Malate, 1 mM GDP. Mitochondria were plated and centrifuged 2,000 g for 20 min to promote adherence to the XF24 V7 cell culture microplate. One millimole of ADP, 4 mM Oligomycin, 6mM FCCP, and 2 mM each of Antimycin A/Rotenone were added during fixed time intervals.

### Plasmids, adenovirus, and lentivirus

The recombinant adenovirues of GFP (control) and p38αAF were generated as previously described in another study [52]. In order to generate the lentivirus for GFP, p38αAF, and MKK6E, the synthesized sequences were inserted into Fugw-vector plasmids, which were PCR from plasmids described previously. The above Fugw plasmids and packaging plasmids PMD2.G and PSPAX were cotransfected into HEK293T cells. The virus particles were collected from supernatant. Titers were determined by using dilution methods and counting the number of GFP-positive colonies using fluorescence microscope. PKA C (CRE) promoters were amplified with primers: PKA C (CRE) KpnI-f: CGG GGT ACC CCG GAC CTA GTC AGA CTT TGG AG; PKA C (CRE) XhoI-r: CCG CTC GAG CGG ATC AGT TTG TCT TGG GGA CT. For the luciferase reporter assay, HEK293T cells were plated in 48-well plates and transfected with PKA C (CRE) reporter constructs and MSCV or CREB vectors (Addgene). The pRL-TK vector (expressing Renilla luciferase) was used to normalize the luciferase activity. Cells were lysed 48 h after transfection, and luciferase activity was measured using a dual-luciferase reporter assay system (Promega).

### ChIP analysis

ChIP assays of SVF-derived matured adipocytes were performed using an EZ Magna ChIP G kit (Minipore) according to the manufacturer’s protocol. Immunoprecipitation was performed using an anti-pCREB (Ser133) antibody or with rabbit IgG (Santa Cruz) as a negative control. Primers used for amplifying CRE2 and CRE4 were CRE2-chip-f, GAT AAG AAG TTA CGA CGG GA; CRE2-chip-r: TCT GAG GAA AGG GTT GAC CT; CRE4-chip-f, GAA GAG TGA CAA AAG GCA CC; and CRE4-chip-r: TAT ATA GCC CCT TGC CGG AG. Immunoprecipitation to identify putative CRE in PKA C promoter was performed using an anti-CREB1 antibody (Abcam) or with rabbit IgG (Santa Cruz) as a negative control. Primers used for amplifying putative CRE in PKA C promoter were PKA C (CRE)-chip-f: AGG GAC AGT GCC TCA AAC CT; PKA C (CRE)-chip-r: TGA CAA GCC TGT ACC AGA GA. The PCR cycle parameters were 95 °C for 5 min, then 30 cycles of 95 °C for 25 s, 60 °C for 30 s, and 72 °C for 30 s, followed by a final extension at 72 °C for 3 min for both the ChIP product and input (represent 0.2%). PCR products were resolved by electrophoresis in a 2% Agarose-gel (Invitrogen).

### Statistical analysis and supplemental information

Data were expressed as means ± SEM. The statistical differences in mean values were assessed by Student *t* test. All experiments were performed at least twice, and representative data are shown. Supporting information includes S1 Table (RT-qPCR primers used in this study), S2 Table (primary antibodies used in this study), 7 Supplemental Figures (S1–S7 Figs), and S1 Data (Excel spreadsheet containing, in separate sheets, the underlying numerical data for all figures).

## Supporting information

S1 Fig Adipocyte-specific deletion of p38α leads to a lean phenotype and a decrease in blood glucose and TG levels, Related to Fig 1. (A-C) Relative p38α and p-p38 protein levels in iBAT (A), iWAT (B), and eWAT (C) of Floxed and Fp38αKO mice (*n* = 3 per group). The densities of p38α and p-p38 bands were quantitated and normalized to Hsp90. See also S1 Data. (D-F) Relative p38α protein levels in liver (D), skeletal muscle (E), and macrophages (F) of Floxed and Fp38αKO mice (*n* = 3 per group). The densities of p38α bands were quantitated and normalized to Hsp90 or tubulin. See also S1 Data. (G) Flow cytometry analysis of F4/80^+^Mac1^+^ macrophages and Gr-1^+^Mac1^+^ neutrophils in the peripheral blood of Floxed and Fp38αKO mice as indicated (*n* = 5 per group). See also S1 Data. (H) LM and FM-to-LM ratio (FM/LM) of Floxed and Fp38αKO mice as indicated (*n* = 11 per group). See also S1 Data. (I-L) Representative HE staining of iWAT (I) and eWAT (K), diameter and cross-sectional area of adipocytes in iWAT (J) and eWAT (L) from Floxed and Fp38αKO mice as indicated. Bars: 100 μm. See also S1 Data. (M) GTT (Floxed: *n* = 8–10; Fp38αKO: *n* = 7–9) in Floxed and Fp38αKO mice. The D-glucose dose was adjusted for LM. AUCs were calculated. See also S1 Data. (N) Glucose (*n* = 10–13 per group) and TG (*n* = 8–9 per group) levels in Floxed and Fp38αKO mice. See also S1 Data. Means ± SEM are shown. *p < 0.05; **p < 0.01; ***p < 0.001. AUC, area under curve; eWAT, epididymal white adipose tissue; FM, fat mass; GTT, glucose tolerance test; HE staining, hematoxylin-eosin staining; iBAT, interscapular brown adipose tissue; iWAT, inguinal white adipose tissue; LM, lean mass; NS, non significant; TG, triglyceride.
(TIF)

S2 Fig Energy expenditure, mitochondrial content, and protein levels of UCP-1 and TH in iBAT in Fp38αKO mice, Related to Fig 2. (A) Change of BW in Floxed and Fp38αKO mice after 2 d of cold exposure (*n* = 6 per group). See also S1 Data. (B) BT of Floxed and Fp38αKO mice exposed to cold for 2 d (*n* = 6 per group). See also S1 Data. (C) iBAT weight and relative iBAT weight to BW ratio (iBAT/BW) of Floxed and Fp38αKO mice exposed to cold for 2 d (*n* = 6 per group). See also S1 Data. (D and E) Representative HE staining (D), diameter and cross-sectional area (E) of iBAT from Floxed and Fp38αKO mice exposed to cold for 2 d. Bars: 100 μm. See also S1 Data. (F and G) Representative HE staining (F), diameter and cross-sectional area (G) of iBAT from Floxed and Fp38αKO mice exposed to cold for 7 d. Bars: 100 μm. See also S1 Data. (H) Relative mitDNA to nuDNA ratio in iBAT from Floxed (*n* = 9) and Fp38αKO (*n* = 6) mice exposed to cold for 2 d. See also S1 Data. (I and J) Relative mRNA levels of CPT1B, CYT C, and TFAM in iBAT from Floxed and Fp38αKO mice maintained at RT (I, *n* = 6 per group) or exposed to cold for 2 d (J, *n* = 8 per group). See also S1 Data. (K) Relative mRNA levels of ATGL, HSL, MGL, LPL, CPT1B, CIDEC, and CD36 in iBAT from Floxed and Fp38αKO mice exposed to cold for 2 d (*n* = 7–8). See also S1 Data. (L) Relative p-ATF2 protein levels in iBAT of Floxed and Fp38αKO mice exposed to cold for 2 d. The densities of p-ATF2 bands were quantitated and normalized to Hsp90 (*n* = 3 per group). See also S1 Data. (M) Percent contribution of the mRNA expression of each p38 isoform to the mRNA expression of total p38 isoforms in mouse iBAT. See also S1 Data. (N and O) Representative western blot of p38β and p38γ in iBAT of Floxed and Fp38αKO mice maintained at RT (N) or exposed to cold for 2 d (O). (P) Representative western blots of TH and UCP-1 in iBAT from Floxed and Fp38αKO mice maintained at RT or exposed to cold for 2 d. (Q) Relative mRNA levels of ACC1, FASN, ME, and CD36 in Floxed (*n* = 6–8) and Fp38αKO (*n* = 8) mice exposed to cold for 4 h. See also S1 Data. (R) NEFA levels in Floxed and Fp38αKO mice maintained at RT (*n* = 10 per group) or exposed to cold for 4 h (*n* = 6–8 per group). See also S1 Data. (S) Relative creatine kinase activity in serum (*n* = 3–4), GAS muscle (*n* = 5–8), and heart (*n* = 6–8) of Floxed and Fp38αKO mice exposed to cold for 4 h. See also S1 Data. Means ± SEM are shown. ACC1, acetyl-CoA carboxylase alpha; ATGL, adipose triglyceride lipase; BT, body temperature; BW, body weight; CIDEC, cell death-inducing DFFA-like effector c; CPT1B, carnitine palmitoyltransferase 1B; CYT C, cytochrome c; FASN, fatty acid synthase; HE staining, hematoxylin-eosin staining; HSL, hormone-sensitive lipase; iBAT, interscapular brown adipose tissue; LPL, lipoprotein lipase; ME, malic enzyme; MGL, monoglyceride lipase; mitDNA, mitochondrial DNA; NEFA, nonesterified fatty acid; NS, not significant; nuDNA, nuclear DNA; RT, room temperature; TFAM, transcription factor A, mitochondrial; TH, tyrosine hydroxylase; UPC-1, uncoupling protein 1.
(TIF)

S3 Fig Increased browning in Fp38αKO mice is independent of sympathetic action, Related to Fig 3. (A) Representative western blots of p-CREB (Ser133) and p38α in iWAT from Floxed and Fp38αKO mice exposed to cold for 2 d. (B) BW of Floxed and Fp38αKO mice maintained at RT prior to CL316,243 injection. See also S1 Data. (C) VO_2_ and VCO_2_ in Floxed and Fp38αKO mice adapted to a cold environment for 7 d (*n* = 4 per group). See also S1 Data. (D and E) Representative HE staining of iWAT (D), diameter and cross-sectional area of adipocytes in iWAT (E) from 5-wk-old Floxed and Fp38αKO mice maintained at RT. Bars: 100 μm. See also S1 Data. (F) Relative mitDNA to nuDNA ratio in unilateral iWAT from 5-wk-old Floxed and Fp38αKO mice maintained at RT (*n* = 9 per group). See also S1 Data. (G and H) Representative HE staining of eWAT (G), diameter and cross-sectional area of adipocytes in eWAT (H) from 5-wk-old Floxed and Fp38αKO mice maintained at RT. Bars: 100 μm. See also S1 Data. (I) Representative western blots of TH in iWAT from 5-wk-old Floxed and Fp38αKO mice maintained at RT. (J) Representative western blots of p38α in iBAT of Ap38αKO mice exposed to cold for 2 d. (K) Representative western blots of p38α and UCP-1 in iWAT of Ap38αKO mice exposed to cold for 2 d. (L and M) Representative UCP-1 and BrdU staining (L) and the percentage of BrdU^+^ adipocytes relative to the total numbers of adipocytes examined (M, *n* = 8–10) in iWAT from Floxed and Fp38αKO mice. These mice were maintained in a cold environment and injected with BrdU twice a day for 7 d before analysis. BrdU^+^ adipocytes were indicated by white dashed triangles. See also S1 Data. (N) Percent contribution of the mRNA expression of each p38 isoform to the mRNA expression of total p38 isoforms in mouse iWAT. See also S1 Data. (O and P) Representative western blot of p38β and p38γ in iWAT of Floxed and Fp38αKO mice maintained at RT (O) or exposed to cold for 2 d (P). Means ± SEM are shown. *p < 0.05; ***p < 0.001. BW, body weight; CREB, cAMP-response element binding protein; HE staining, hematoxylin-eosin staining; iBAT, interscapular brown adipose tissue; iWAT, inguinal white adipose tissue; mitDNA, mitochondrial DNA; NS, not significant; nuDNA, nuclear DNA; RT, room temperature; TH, tyrosine hydroxylase; UCP-1, uncoupling protein 1; VO_2_, oxygen consumption; VCO_2_, carbon dioxide production.
(TIF)

S4 Fig p38α deficiency in adipose tissues improves fatty liver, Related to Fig 4. (A-C) Weight of iBAT (A), GAS muscle (B), and liver (C) from Floxed (*n* = 5–8) and Fp38αKO (*n* = 6–7) mice after HFD feeding. See also S1 Data. (D and E) Representative HE staining (d, bars: 100 μm) and Oil Red O staining (e, bars: [top] 100 μm; [bottom] 50 μm) of liver from Floxed and Fp38αKO mice after HFD feeding. (F and G) Representative HE staining of eWAT (F), diameter and cross-sectional area of adipocytes in eWAT (G) from HFD-fed Floxed and Fp38αKO mice exposed to cold for 2 d. Bars: 100 μm. See also S1 Data. (H and I) VO_2_ and VCO_2_ in Floxed (*n* = 8) and Fp38αKO (*n* = 6) mice after HFD feeding. The values were normalized by BW (h) or by LM (I), respectively. See also S1 Data. (J and K) Representative CD68 staining of iWAT (J) and relative amount of CD68^+^ cells per field in iWAT (K, *n* = 4 per group) from HFD-fed Floxed and Fp38αKO mice exposed to cold for 2 d. See also S1 Data. (L and M) Relative mRNA levels of ARG-1, MRC-1, and FIZZ1(L) and COX2, IFN-γ, and CCL-2 (M) in iWAT from HFD-fed Floxed and Fp38αKO mice exposed to cold for 2 d (*n* = 4–6 per group). See also S1 Data. (N and O) Representative UCP-1 and BrdU staining of iWAT (N), the percentage of BrdU^+^ adipocytes relative to the total numbers of adipocytes examined, and the percentage of BrdU^-^UCP-1^+^ adipocytes relative to the total numbers of UCP-1^+^ adipocytes examined in iWAT (O, *n* = 4 per group) from HFD-fed Floxed and Fp38αKO mice. These mice were maintained in a cold environment and injected with BrdU twice a day for 7 d before analysis. BrdU^+^ adipocytes were indicated by white dashed triangles. See also S1 Data. Means ± SEM are shown. *p < 0.05; **p < 0.01. ARG-1, arginase 1; BW, body weight; CCL-2, C-C motif chemokine ligand 2; COX2, cytochrome c oxidase subunit II; eWAT, epididymal white adipose tissue; GAS, gastrocnemius; HE staining, hematoxylin-eosin staining; HFD, high-fat diet; iBAT, interscapular brown adipose tissue; IFN-γ, interferon gamma; iWAT, inguinal white adipose tissue; LM, lean mass; NS, not significant; UCP-1, uncoupling protein 1; VCO_2_, carbon dioxide production; VO_2_, oxygen consumption.
(TIF)

S5 Fig Pharmaceutical inhibition of p38α decreases glucose levels and prevents weight gain, Related to Fig 5. (A and B) Relative weight of iBAT (A, *n* = 5 per group) and liver (B, *n* = 5 per group) of C57BL/6J mice received 4 wk of SB203580 treatment. See also S1 Data. (C and D) Glucose levels of C57BL/6J mice received 4 wk of SB203580 treatment. These mice were maintained at RT (C, *n* = 13 per group) or exposed to cold for 2 d (D, *n* = 5 per group). See also S1 Data. (E) Ex vivo-measured ^18^FDG uptake in GAS muscle to tissue weight ratio by γ counter (*n* = 3 per group). See also S1 Data. (F) Relative mRNA levels of PGC1α, FOXO1, MB, TNNI1, MHC I, MHC IIa, MHC IIb, and MHC IIX in GAS muscle from SB203580-treated C57BL/6J mice at 2 d postinjection (*n* = 9–15 per group). See also S1 Data. (G) Change of BW of *db/db* mice after 3 wk of SB203580 treatment (*n* = 5 per group). See also S1 Data. (H) BW of *db/db* mice before and after treatment with SB203580 for 3 wk (*n* = 5 per group). See also S1 Data. (I and J) Representative CD68 staining of iWAT (I) and relative amount of CD68^+^ cells per field in iWAT (J, *n* = 3 per group) from *db/db* mice after 3 wk of SB203580 treatment. See also S1 Data. (K and L) Relative mRNA levels of ARG-1, MRC-1, FIZZ1, and YM-1 (K; *n* = 8–10 per group) and COX2, IFN-γ, and CCL-2 (L; *n* = 6–9 per group) in iWAT of *db/db* mice after 3 wk of SB203580 treatment. See also S1 Data. Means ± SEM are shown. *p < 0.05; **p < 0.01. ARG-1, arginase 1; BW, body weight; CCL-2, C-C motif chemokine ligand 2; COX2, cytochrome c oxidase subunit II; FOXO1, forkhead box O1; GAS, gastrocnemius; iBAT, interscapular brown adipose tissue; IFN-γ, interferon gamma; iWAT, inguinal white adipose tissue; MB, myoglobin; MHC I, myosin heavy chain, class I; MHC IIX, myosin, heavy polypeptide 1, skeletal muscle, adult; NS, not significant; PGC1α, peroxisome proliferative activated receptor gamma coactivator 1α; RT, room temperature; TNNI1, troponin I, skeletal, slow 1.
(TIF)

S6 Fig The effect of p38α inhibition or deficiency on adipocytes is cell autonomous and cell-type specific, Related to Fig 6. (A and B) Relative mRNA levels of p38α (A, *n* = 6–8 per group) and protein levels of p-p38 (B) in iWAT of C57BL/6J mice after Ad-p38αAF infection. See also S1 Data. (C) Glucose and TG levels of C57BL/6J mice after Ad-p38αAF infection (*n* = 4–8 per group). Mice were exposed to cold for 2 d before glucose and TG measurement. See also S1 Data. (D) Relative mRNA levels of UCP-1 and DIO2 in matured 3T3L1 adipocytes treated with SB203580 for 4 h (*n* = 4 per group). See also S1 Data. (E) Relative mRNA levels of UCP-1, PGC1α, PRDM16, and CIDEA in BAC cells treated with SB203580 (*n* = 6 per group). See also S1 Data. (f) Relative mRNA levels of UCP-1, PGC1α, DIO2, ELVOL3, COX8B, and CIDEA in iBAT-SVF-derived matured adipocytes treated with SB203580 for 4 h (*n* = 3–6 per group). See also S1 Data. (G) Percent contribution of the mRNA expression of each p38 isoform to the mRNA expression of total p38 isoforms in matured adipocytes derived from iBAT-SVF or iWAT-SVF. See also S1 Data. (H) Relative mRNA levels of UCP-1, PRDM16, DIO2, ELVOL3, COX8B, and CIDEA in iBAT-SVF-derived matured adipocytes treated with p38α-specific inhibitor (p38αMAPK-IN-1) for 4 h (*n* = 3 per group). See also S1 Data. (I) Relative mRNA levels of p38α in matured 3T3L1 adipocytes after infection with Lenti-p38αAF (*n* = 4 per group). See also S1 Data. (J) Relative mRNA levels of UCP-1, PGC1α, and COX8B in matured 3T3L1 adipocytes after infection with Lenti-p38αAF (*n* = 3 per group). See also S1 Data. (K) Representative Oil Red O staining of matured 3T3L1 adipocytes after Lenti-p38αAF infection. Bars: 25 μm. (L) Relative mRNA levels of p38α in iWAT-SVF-derived matured adipocytes infected with Lenti-p38αAF (*n* = 3 per group). See also S1 Data. (M) Representative Oil Red O staining of adipocytes derived from iWAT-SVF of Floxed and Fp38αKO mice. Bars: 50 μm. The picture for the dishes after Oil Red O staining is shown on the left. (N) Imaging of adipocytes derived from iWAT-SVF of Floxed and Fp38αKO mice after differentiation. Lipid droplets were stained with antibodies against perilipin. Bars: 10 μm. (O) Relative mRNA levels of UCP-1, PGC1α, PRDM16, DIO2, ELVOL3, COX8B, and CIDEA in matured adipocytes derived from iBAT-SVF of Floxed and Fp38αKO mice (*n* = 3 per group). See also S1 Data. (P) Relative mRNA levels of UCP-1 and DIO2 in matured adipocytes derived from WAT-SVF of Ap38αKO mice (*n* = 4 per group). See also S1 Data. Means ± SEM are shown. *p < 0.05; **p < 0.01; ***p < 0.001. CIDEA, cell death-inducing DNA fragmentation factor, alpha subunit-like effector A; COX8B, cytochrome c oxidase subunit 8B; DIO2, deiodinase 2; ELVOL3, elongation of very long chain fatty acids (FEN1/Elo2, SUR4/Elo3, yeast)-like 3; iBAT, interscapular brown adipose tissue; iWAT, inguinal white adipose tissue; Lenti-p38αAF, lentivirus expressing p38αAF; MAPK, mitogen-activated protein kinase; PGC1α, peroxisome proliferative activated receptor gamma coactivator 1α; PRDM16, positive regulatory domain containing 16; SVF, stromal vascular fraction; TG, triglyceride; UCP-1, uncoupling protein 1; WAT, white adipose tissue.
(TIF)

S7 Fig PKA/CREB pathway mediates the effect of p38α deficiency in WAT, Related to Fig 7. (A and B) Representative western blots of p-CREB (Ser133) in matured adipocytes derived from iWAT-SVF of Floxed and Fp38αKO mice (A) or iWAT-SVF-derived matured adipocytes infected with Lenti-p38αAF (B) after FSK treatment for indicated time. (C and D) Representative results of ChIP analysis of p-CREB enrichment on CRE2 (C) and CRE4 (D) in the UCP-1 enhancer, respectively, in matured adipocytes derived from iWAT-SVF of Floxed and Fp38αKO mice. (E) Representative p-CREB (Ser133) and PKA C staining of iWAT from 5-wk-old Floxed and Fp38αKO mice maintained at RT at low (left) and high (right) magnification as indicated. Bars: (left 4 panels) 100 μm; (right 4 panels) 50 μm. (F) Phosphorylated PKA substrates in matured 3T3L1 adipocytes after infection with Lenti-p38αAF and FSK treatment. (G) Representative western blots of p-CREB (Ser133) in BAC cells treated with SB203580. (H) Representative western blots of p-ATF2, p-CREB (Ser133), and UCP-1 in matured adipocytes from iBAT-SVF of Floxed and Fp38αKO mice. (I) Representative western blots of p-CREB (Ser133) in iBAT from Floxed and Fp38αKO mice maintained at RT. (J) Representative western blots of p-ATF2, PKA C, p-PKA C, and p-CREB (Ser133) in matured iWAT-SVF-derived adipocytes treated with SB203580. ATF2, activating transcription factor 2; BAC, brown adipocyte cell line; ChIP, chromatin immunoprecipitation; CRE, cAMP response element; CREB, cAMP-response element binding protein; FSK, forskolin; iWAT, inguinal white adipose tissue; Lenti-p38αAF, lentivirus expressing p38αAF; PKA, protien kinase A; PKA C, PKA catalytic subunit; RT, room temperature; SVF, stromal vascular fraction; UCP-1, uncoupling protein 1; WAT, white adipose tissue.
(TIF)

S1 Table RT-qPCR primers used in this study. RT-qPCR, quantitative reverse transcription PCR.
(DOCX)

S2 Table Primary antibodies used in this study. (DOCX)

S1 Data Numerical data used in figures. (XLSX)

## Abbreviations

ADP: adenosine diphosphate
Ad-p38αAF: adenovirus expressing p38αAF
ASK1: apoptosis signal-regulating kinase 1
ATF2: activating transcription factor 2
ATGL: adipose triglyceride lipase
AUC: area under curve
BAC: brown adipocyte cell line
BAT: brown adipose tissue
BT: body temperature
BW: body weight
CAS: Chinese Academy of Sciences
ChIP: chromatin immunoprecipitation
CRE: cAMP response element
Cre: recombinase
CRE2: cAMP response element 2
CRE4: cAMP response element 4
CREB: cAMP-response element binding protein
CT: computed tomography
DIO2: deiodinase 2
ELVOL3: elongation of very long chain fatty acids (FEN1/Elo2, SUR4/Elo3, yeast)-like 3
eWAT: epididymal white adipose tissue
FCCP: carbonyl cyanide 4-(trifluoromethoxy)phenylhydrazone
FM: fat mass
Fp38αKO: adipocyte-specific p38α knockout
GAS: gastrocnemius
GTT: glucose tolerance test
HE: staining, hematoxylin-eosin staining
HFD: high-fat diet
HSL: hormone-sensitive lipase
iBAT: interscapular brown adipose tissue
INS: Institute for Nutritional Sciences
ITT: insulin tolerance test
iWAT: inguinal white adipose tissue
Lenti-MKK6E: lentivirus expressing a constitutive active mutant of a mitogen-activated protein kinase kinase
Lenti-p38αAF: lentivirus expressing p38αAF
LM: lean mass
MAPK: mitogen-activated protein kinase
MGL: monoglyceride lipase
mitDNA: mitochondrial DNA
ND: not detectable
NEFA: nonesterified fatty acid
NS: not significant
nuDNA: nuclear DNA
OCR: oxygen consumption rate
PET: positron emission tomography
PGC1α: peroxisome proliferative activated receptor gamma coactivator 1α
PKA C: PKA catalytic subunit
PKA: protien kinase A
PRDM16: positive regulatory domain containing 16
RT: room temperature
SIBS: Shanghai Institutes for Biological Sciences
SVF: stromal vascular fraction
TG: triglyceride
TH: tyrosine hydroxylase
UCP-1: uncoupling protein 1
WAT: white adipose tissue
