# Supplementary figures and images for: Metabolic benefits of inhibition of p38α in white adipose tissue in obesity
Source: PLoS Biol. 2018 May 11;16(5):e2004225. doi: 10.1371/journal.pbio.2004225 (PMC5965899; doi:10.1371/journal.pbio.2004225)

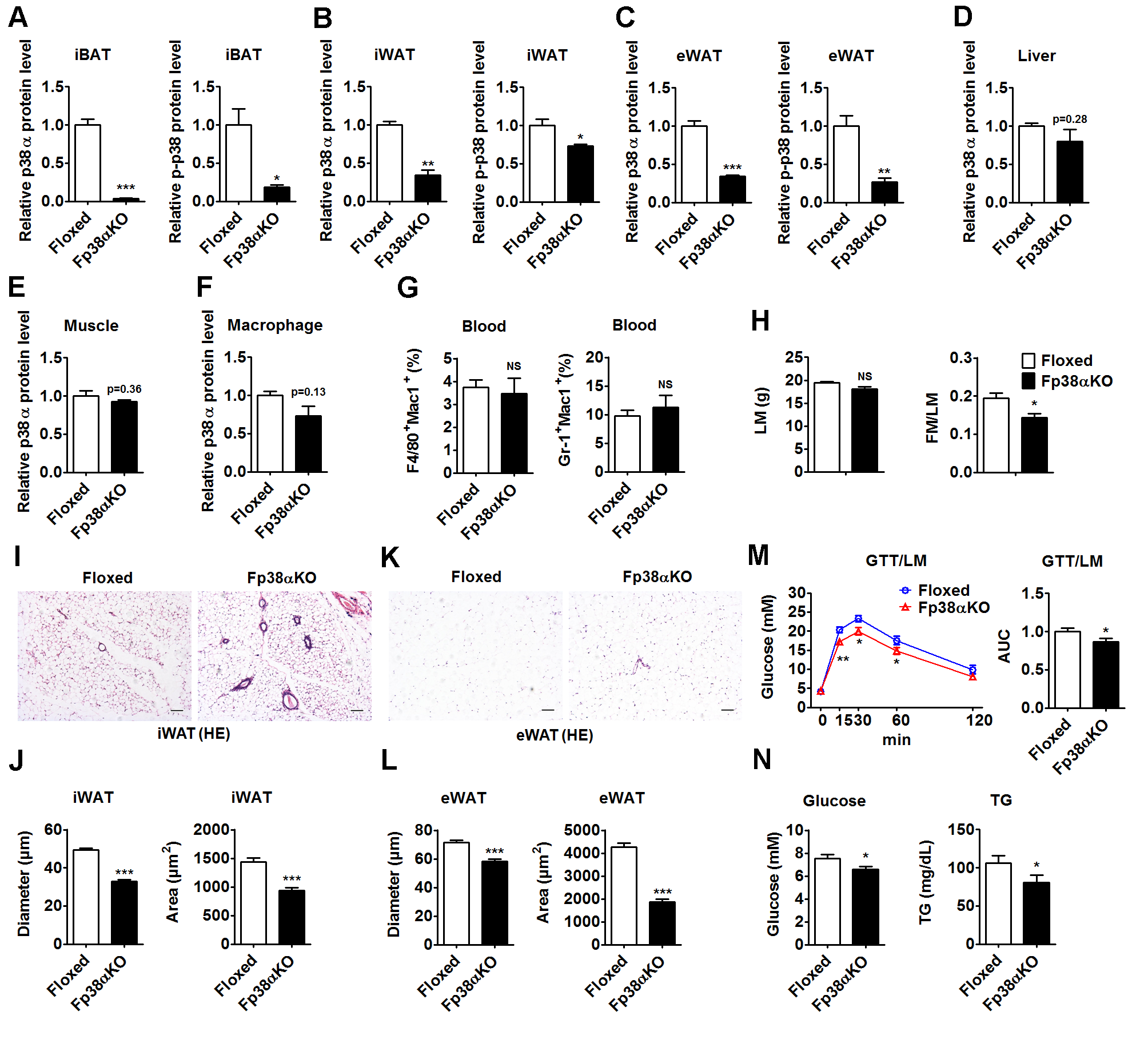

Supplement: S1 Fig — (A-C) Relative p38α and p-p38 protein levels in iBAT (A), iWAT (B), and eWAT (C) of Floxed and Fp38αKO mice (n = 3 per group). The densities of p38α and p-p38 bands were quantitated and normalized to Hsp90. See also S1 Data. (D-F) Relative p38α protein levels in liver (D), skeletal muscle (E), and macrophages (F) of Floxed and Fp38αKO mice (n = 3 per group). The densities of p38α bands were quantitated and normalized to Hsp90 or tubulin. See also S1 Data. (G) Flow cytometry analysis of F4/80+Mac1+ macrophages and Gr-1+Mac1+ neutrophils in the peripheral blood of Floxed and Fp38αKO mice as indicated (n = 5 per group). See also S1 Data. (H) LM and FM-to-LM ratio (FM/LM) of Floxed and Fp38αKO mice as indicated (n = 11 per group). See also S1 Data. (I-L) Representative HE staining of iWAT (I) and eWAT (K), diameter and cross-sectional area of adipocytes in iWAT (J) and eWAT (L) from Floxed and Fp38αKO mice as indicated. Bars: 100 μm. See also S1 Data. (M) GTT (Floxed: n = 8–10; Fp38αKO: n = 7–9) in Floxed and Fp38αKO mice. The D-glucose dose was adjusted for LM. AUCs were calculated. See also S1 Data. (N) Glucose (n = 10–13 per group) and TG (n = 8–9 per group) levels in Floxed and Fp38αKO mice. See also S1 Data. Means ± SEM are shown. *p < 0.05; **p < 0.01; ***p < 0.001. AUC, area under curve; eWAT, epididymal white adipose tissue; FM, fat mass; GTT, glucose tolerance test; HE staining, hematoxylin-eosin staining; iBAT, interscapular brown adipose tissue; iWAT, inguinal white adipose tissue; LM, lean mass; NS, non significant; TG, triglyceride. (TIF) [file pbio.2004225.s001.tif]

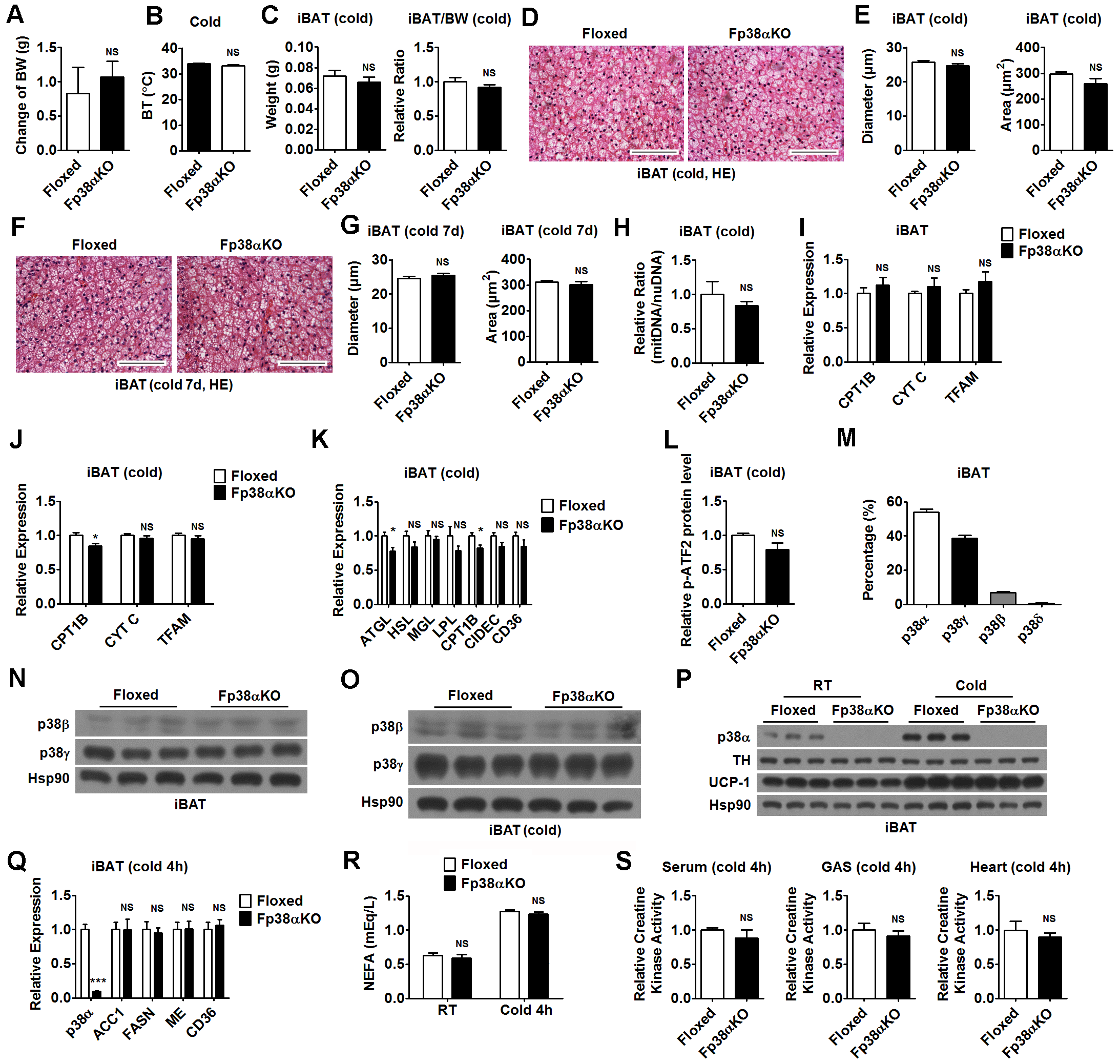

Supplement: S2 Fig — (A) Change of BW in Floxed and Fp38αKO mice after 2 d of cold exposure (n = 6 per group). See also S1 Data. (B) BT of Floxed and Fp38αKO mice exposed to cold for 2 d (n = 6 per group). See also S1 Data. (C) iBAT weight and relative iBAT weight to BW ratio (iBAT/BW) of Floxed and Fp38αKO mice exposed to cold for 2 d (n = 6 per group). See also S1 Data. (D and E) Representative HE staining (D), diameter and cross-sectional area (E) of iBAT from Floxed and Fp38αKO mice exposed to cold for 2 d. Bars: 100 μm. See also S1 Data. (F and G) Representative HE staining (F), diameter and cross-sectional area (G) of iBAT from Floxed and Fp38αKO mice exposed to cold for 7 d. Bars: 100 μm. See also S1 Data. (H) Relative mitDNA to nuDNA ratio in iBAT from Floxed (n = 9) and Fp38αKO (n = 6) mice exposed to cold for 2 d. See also S1 Data. (I and J) Relative mRNA levels of CPT1B, CYT C, and TFAM in iBAT from Floxed and Fp38αKO mice maintained at RT (I, n = 6 per group) or exposed to cold for 2 d (J, n = 8 per group). See also S1 Data. (K) Relative mRNA levels of ATGL, HSL, MGL, LPL, CPT1B, CIDEC, and CD36 in iBAT from Floxed and Fp38αKO mice exposed to cold for 2 d (n = 7–8). See also S1 Data. (L) Relative p-ATF2 protein levels in iBAT of Floxed and Fp38αKO mice exposed to cold for 2 d. The densities of p-ATF2 bands were quantitated and normalized to Hsp90 (n = 3 per group). See also S1 Data. (M) Percent contribution of the mRNA expression of each p38 isoform to the mRNA expression of total p38 isoforms in mouse iBAT. See also S1 Data. (N and O) Representative western blot of p38β and p38γ in iBAT of Floxed and Fp38αKO mice maintained at RT (N) or exposed to cold for 2 d (O). (P) Representative western blots of TH and UCP-1 in iBAT from Floxed and Fp38αKO mice maintained at RT or exposed to cold for 2 d. (Q) Relative mRNA levels of ACC1, FASN, ME, and CD36 in Floxed (n = 6–8) and Fp38αKO (n = 8) mice exposed to cold for 4 h. See also S1 Data. (R) NEFA levels in Floxed and Fp38αKO mice [file pbio.2004225.s002.tif]

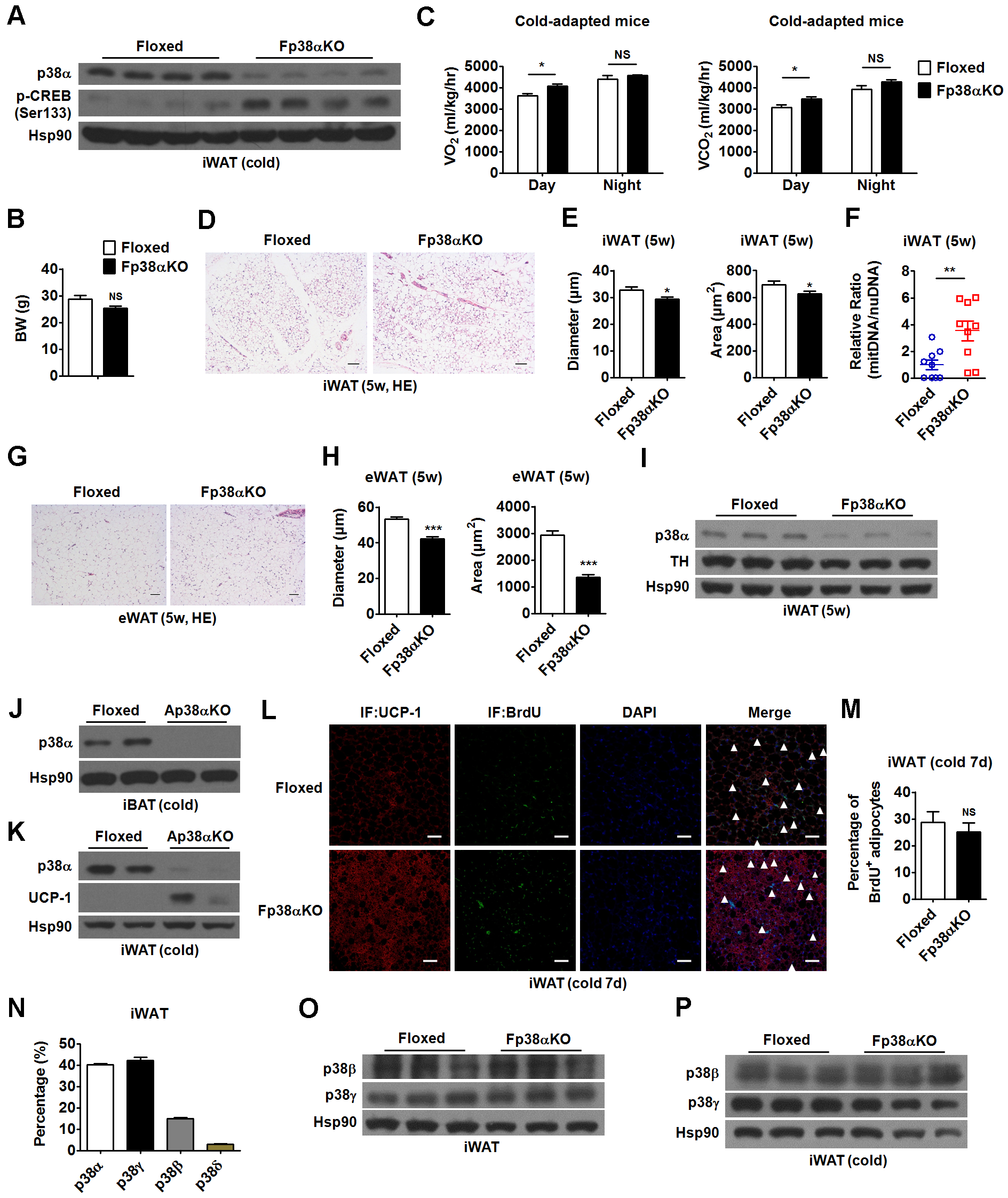

Supplement: S3 Fig — (A) Representative western blots of p-CREB (Ser133) and p38α in iWAT from Floxed and Fp38αKO mice exposed to cold for 2 d. (B) BW of Floxed and Fp38αKO mice maintained at RT prior to CL316,243 injection. See also S1 Data. (C) VO2 and VCO2 in Floxed and Fp38αKO mice adapted to a cold environment for 7 d (n = 4 per group). See also S1 Data. (D and E) Representative HE staining of iWAT (D), diameter and cross-sectional area of adipocytes in iWAT (E) from 5-wk-old Floxed and Fp38αKO mice maintained at RT. Bars: 100 μm. See also S1 Data. (F) Relative mitDNA to nuDNA ratio in unilateral iWAT from 5-wk-old Floxed and Fp38αKO mice maintained at RT (n = 9 per group). See also S1 Data. (G and H) Representative HE staining of eWAT (G), diameter and cross-sectional area of adipocytes in eWAT (H) from 5-wk-old Floxed and Fp38αKO mice maintained at RT. Bars: 100 μm. See also S1 Data. (I) Representative western blots of TH in iWAT from 5-wk-old Floxed and Fp38αKO mice maintained at RT. (J) Representative western blots of p38α in iBAT of Ap38αKO mice exposed to cold for 2 d. (K) Representative western blots of p38α and UCP-1 in iWAT of Ap38αKO mice exposed to cold for 2 d. (L and M) Representative UCP-1 and BrdU staining (L) and the percentage of BrdU+ adipocytes relative to the total numbers of adipocytes examined (M, n = 8–10) in iWAT from Floxed and Fp38αKO mice. These mice were maintained in a cold environment and injected with BrdU twice a day for 7 d before analysis. BrdU+ adipocytes were indicated by white dashed triangles. See also S1 Data. (N) Percent contribution of the mRNA expression of each p38 isoform to the mRNA expression of total p38 isoforms in mouse iWAT. See also S1 Data. (O and P) Representative western blot of p38β and p38γ in iWAT of Floxed and Fp38αKO mice maintained at RT (O) or exposed to cold for 2 d (P). Means ± SEM are shown. *p < 0.05; ***p < 0.001. BW, body weight; CREB, cAMP-response element binding protein; HE staining, hematoxylin-eosin staining; i [file pbio.2004225.s003.tif]

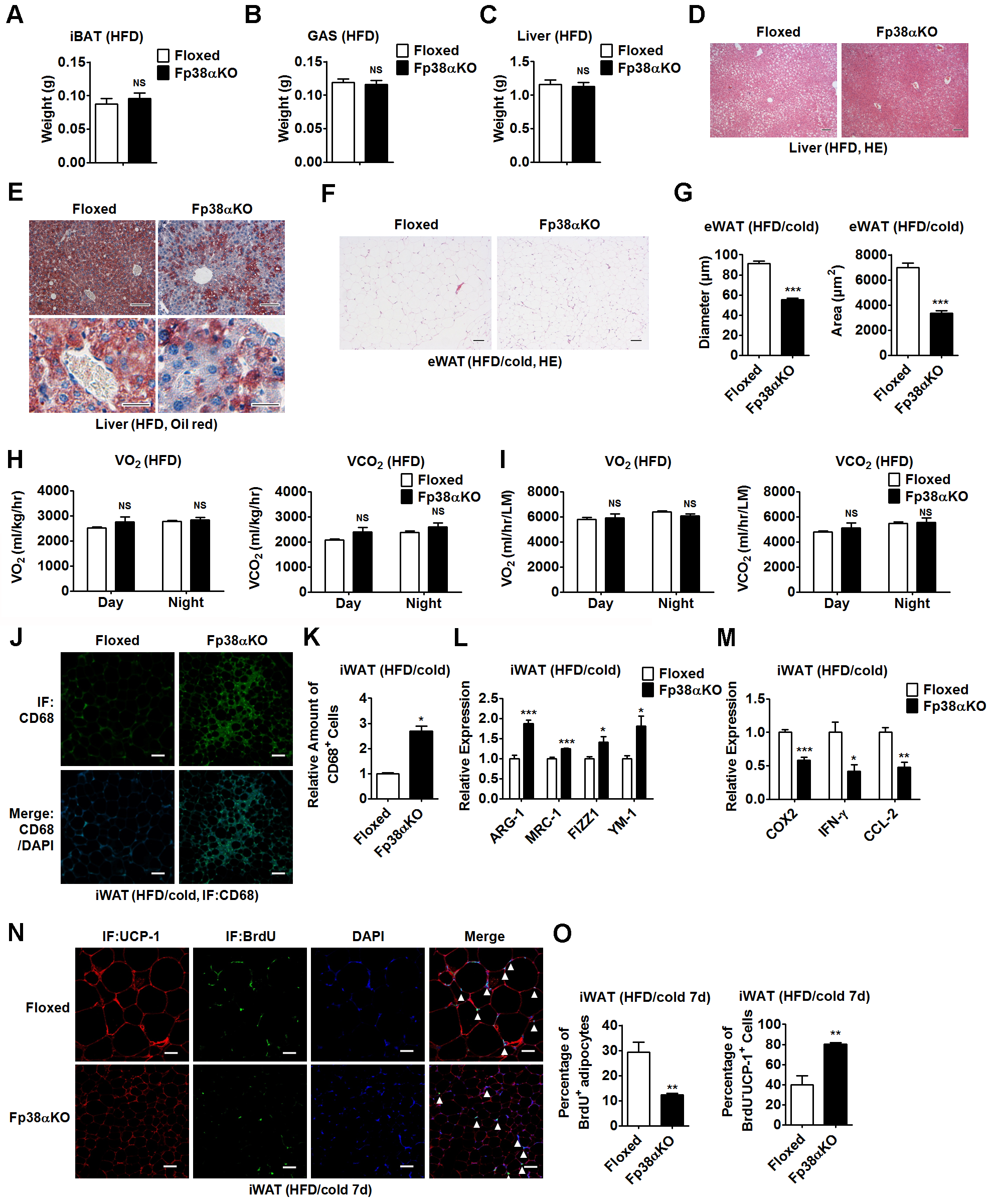

Supplement: S4 Fig — (A-C) Weight of iBAT (A), GAS muscle (B), and liver (C) from Floxed (n = 5–8) and Fp38αKO (n = 6–7) mice after HFD feeding. See also S1 Data. (D and E) Representative HE staining (d, bars: 100 μm) and Oil Red O staining (e, bars: [top] 100 μm; [bottom] 50 μm) of liver from Floxed and Fp38αKO mice after HFD feeding. (F and G) Representative HE staining of eWAT (F), diameter and cross-sectional area of adipocytes in eWAT (G) from HFD-fed Floxed and Fp38αKO mice exposed to cold for 2 d. Bars: 100 μm. See also S1 Data. (H and I) VO2 and VCO2 in Floxed (n = 8) and Fp38αKO (n = 6) mice after HFD feeding. The values were normalized by BW (h) or by LM (I), respectively. See also S1 Data. (J and K) Representative CD68 staining of iWAT (J) and relative amount of CD68+ cells per field in iWAT (K, n = 4 per group) from HFD-fed Floxed and Fp38αKO mice exposed to cold for 2 d. See also S1 Data. (L and M) Relative mRNA levels of ARG-1, MRC-1, and FIZZ1(L) and COX2, IFN-γ, and CCL-2 (M) in iWAT from HFD-fed Floxed and Fp38αKO mice exposed to cold for 2 d (n = 4–6 per group). See also S1 Data. (N and O) Representative UCP-1 and BrdU staining of iWAT (N), the percentage of BrdU+ adipocytes relative to the total numbers of adipocytes examined, and the percentage of BrdU-UCP-1+ adipocytes relative to the total numbers of UCP-1+ adipocytes examined in iWAT (O, n = 4 per group) from HFD-fed Floxed and Fp38αKO mice. These mice were maintained in a cold environment and injected with BrdU twice a day for 7 d before analysis. BrdU+ adipocytes were indicated by white dashed triangles. See also S1 Data. Means ± SEM are shown. *p < 0.05; **p < 0.01. ARG-1, arginase 1; BW, body weight; CCL-2, C-C motif chemokine ligand 2; COX2, cytochrome c oxidase subunit II; eWAT, epididymal white adipose tissue; GAS, gastrocnemius; HE staining, hematoxylin-eosin staining; HFD, high-fat diet; iBAT, interscapular brown adipose tissue; IFN-γ, interferon gamma; iWAT, inguinal white adipose tissue; LM, lean mass; [file pbio.2004225.s004.tif]

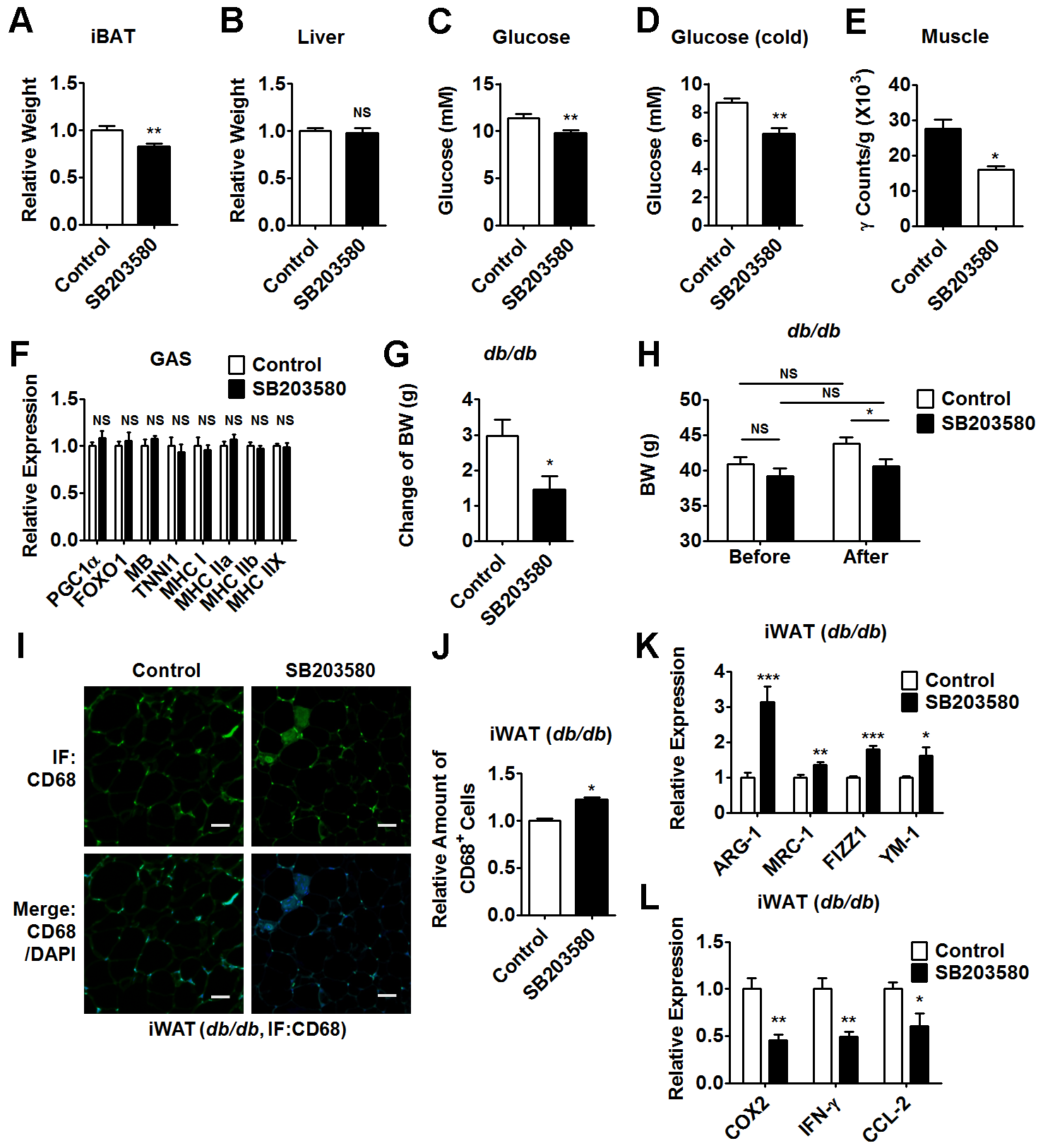

Supplement: S5 Fig — (A and B) Relative weight of iBAT (A, n = 5 per group) and liver (B, n = 5 per group) of C57BL/6J mice received 4 wk of SB203580 treatment. See also S1 Data. (C and D) Glucose levels of C57BL/6J mice received 4 wk of SB203580 treatment. These mice were maintained at RT (C, n = 13 per group) or exposed to cold for 2 d (D, n = 5 per group). See also S1 Data. (E) Ex vivo-measured 18FDG uptake in GAS muscle to tissue weight ratio by γ counter (n = 3 per group). See also S1 Data. (F) Relative mRNA levels of PGC1α, FOXO1, MB, TNNI1, MHC I, MHC IIa, MHC IIb, and MHC IIX in GAS muscle from SB203580-treated C57BL/6J mice at 2 d postinjection (n = 9–15 per group). See also S1 Data. (G) Change of BW of db/db mice after 3 wk of SB203580 treatment (n = 5 per group). See also S1 Data. (H) BW of db/db mice before and after treatment with SB203580 for 3 wk (n = 5 per group). See also S1 Data. (I and J) Representative CD68 staining of iWAT (I) and relative amount of CD68+ cells per field in iWAT (J, n = 3 per group) from db/db mice after 3 wk of SB203580 treatment. See also S1 Data. (K and L) Relative mRNA levels of ARG-1, MRC-1, FIZZ1, and YM-1 (K; n = 8–10 per group) and COX2, IFN-γ, and CCL-2 (L; n = 6–9 per group) in iWAT of db/db mice after 3 wk of SB203580 treatment. See also S1 Data. Means ± SEM are shown. *p < 0.05; **p < 0.01. ARG-1, arginase 1; BW, body weight; CCL-2, C-C motif chemokine ligand 2; COX2, cytochrome c oxidase subunit II; FOXO1, forkhead box O1; GAS, gastrocnemius; iBAT, interscapular brown adipose tissue; IFN-γ, interferon gamma; iWAT, inguinal white adipose tissue; MB, myoglobin; MHC I, myosin heavy chain, class I; MHC IIX, myosin, heavy polypeptide 1, skeletal muscle, adult; NS, not significant; PGC1α, peroxisome proliferative activated receptor gamma coactivator 1α; RT, room temperature; TNNI1, troponin I, skeletal, slow 1. (TIF) [file pbio.2004225.s005.tif]

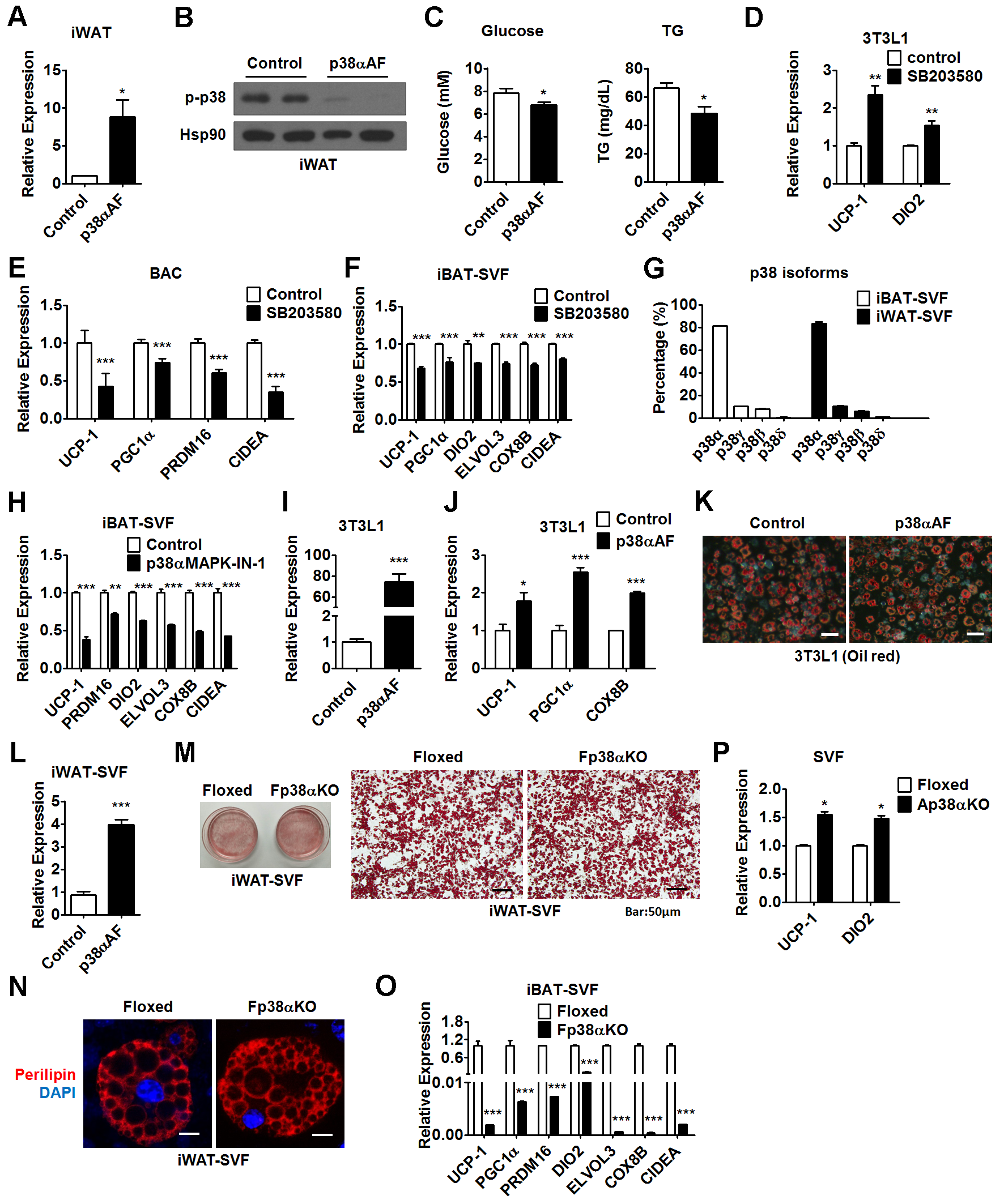

Supplement: S6 Fig — (A and B) Relative mRNA levels of p38α (A, n = 6–8 per group) and protein levels of p-p38 (B) in iWAT of C57BL/6J mice after Ad-p38αAF infection. See also S1 Data. (C) Glucose and TG levels of C57BL/6J mice after Ad-p38αAF infection (n = 4–8 per group). Mice were exposed to cold for 2 d before glucose and TG measurement. See also S1 Data. (D) Relative mRNA levels of UCP-1 and DIO2 in matured 3T3L1 adipocytes treated with SB203580 for 4 h (n = 4 per group). See also S1 Data. (E) Relative mRNA levels of UCP-1, PGC1α, PRDM16, and CIDEA in BAC cells treated with SB203580 (n = 6 per group). See also S1 Data. (f) Relative mRNA levels of UCP-1, PGC1α, DIO2, ELVOL3, COX8B, and CIDEA in iBAT-SVF-derived matured adipocytes treated with SB203580 for 4 h (n = 3–6 per group). See also S1 Data. (G) Percent contribution of the mRNA expression of each p38 isoform to the mRNA expression of total p38 isoforms in matured adipocytes derived from iBAT-SVF or iWAT-SVF. See also S1 Data. (H) Relative mRNA levels of UCP-1, PRDM16, DIO2, ELVOL3, COX8B, and CIDEA in iBAT-SVF-derived matured adipocytes treated with p38α-specific inhibitor (p38αMAPK-IN-1) for 4 h (n = 3 per group). See also S1 Data. (I) Relative mRNA levels of p38α in matured 3T3L1 adipocytes after infection with Lenti-p38αAF (n = 4 per group). See also S1 Data. (J) Relative mRNA levels of UCP-1, PGC1α, and COX8B in matured 3T3L1 adipocytes after infection with Lenti-p38αAF (n = 3 per group). See also S1 Data. (K) Representative Oil Red O staining of matured 3T3L1 adipocytes after Lenti-p38αAF infection. Bars: 25 μm. (L) Relative mRNA levels of p38α in iWAT-SVF-derived matured adipocytes infected with Lenti-p38αAF (n = 3 per group). See also S1 Data. (M) Representative Oil Red O staining of adipocytes derived from iWAT-SVF of Floxed and Fp38αKO mice. Bars: 50 μm. The picture for the dishes after Oil Red O staining is shown on the left. (N) Imaging of adipocytes derived from iWAT-SVF of Floxed and Fp38αKO mice after differentia [file pbio.2004225.s006.tif]

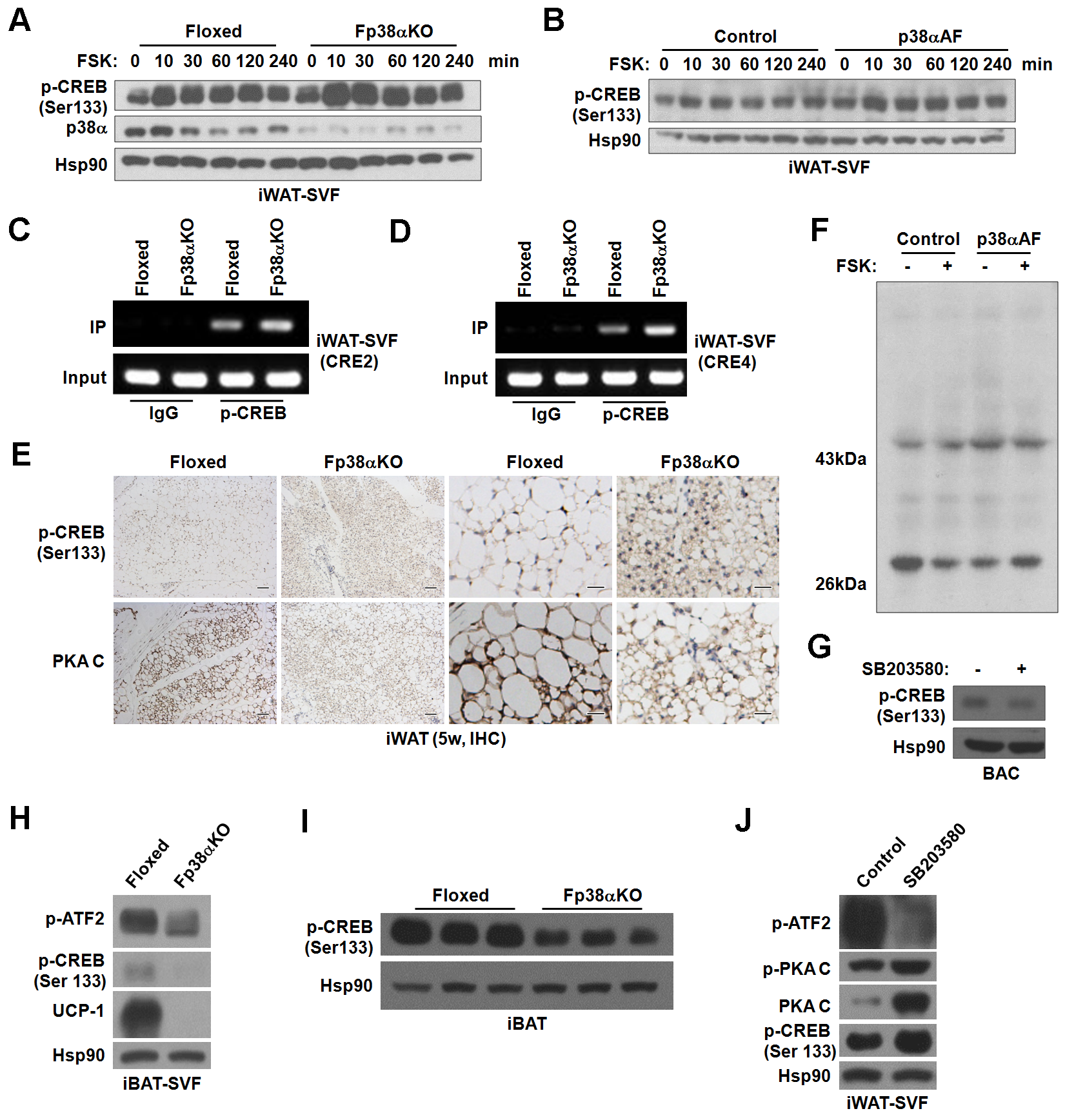

Supplement: S7 Fig — (A and B) Representative western blots of p-CREB (Ser133) in matured adipocytes derived from iWAT-SVF of Floxed and Fp38αKO mice (A) or iWAT-SVF-derived matured adipocytes infected with Lenti-p38αAF (B) after FSK treatment for indicated time. (C and D) Representative results of ChIP analysis of p-CREB enrichment on CRE2 (C) and CRE4 (D) in the UCP-1 enhancer, respectively, in matured adipocytes derived from iWAT-SVF of Floxed and Fp38αKO mice. (E) Representative p-CREB (Ser133) and PKA C staining of iWAT from 5-wk-old Floxed and Fp38αKO mice maintained at RT at low (left) and high (right) magnification as indicated. Bars: (left 4 panels) 100 μm; (right 4 panels) 50 μm. (F) Phosphorylated PKA substrates in matured 3T3L1 adipocytes after infection with Lenti-p38αAF and FSK treatment. (G) Representative western blots of p-CREB (Ser133) in BAC cells treated with SB203580. (H) Representative western blots of p-ATF2, p-CREB (Ser133), and UCP-1 in matured adipocytes from iBAT-SVF of Floxed and Fp38αKO mice. (I) Representative western blots of p-CREB (Ser133) in iBAT from Floxed and Fp38αKO mice maintained at RT. (J) Representative western blots of p-ATF2, PKA C, p-PKA C, and p-CREB (Ser133) in matured iWAT-SVF-derived adipocytes treated with SB203580. ATF2, activating transcription factor 2; BAC, brown adipocyte cell line; ChIP, chromatin immunoprecipitation; CRE, cAMP response element; CREB, cAMP-response element binding protein; FSK, forskolin; iWAT, inguinal white adipose tissue; Lenti-p38αAF, lentivirus expressing p38αAF; PKA, protien kinase A; PKA C, PKA catalytic subunit; RT, room temperature; SVF, stromal vascular fraction; UCP-1, uncoupling protein 1; WAT, white adipose tissue. (TIF) [file pbio.2004225.s007.tif]
