# Supplementary material for: Metabolic benefits of inhibition of p38α in white adipose tissue in obesity
Source: PLoS Biol. 2018 May 11;16(5):e2004225. doi: 10.1371/journal.pbio.2004225 (PMC5965899; doi:10.1371/journal.pbio.2004225)
Supplement: S1 Table — RT-qPCR, quantitative reverse transcription PCR. (DOCX) [file pbio.2004225.s008.docx]

**S1 Table. RT-qPCR primers**

| 18S F | ACCGCAGCTAGGAATAATGGA |
| --- | --- |
| 18S R | CAAATGCTTTCGCTCTGGTC |
| UCP-1 F | ACTGCCACACCTCCAGTCATT |
| UCP-1 R | CTTTGCCTCACTCAGGATTGG |
| PGC1α F | TGATGTGAATGACTTGGATACAGACA |
| PGC1α R | GCTCATTGTTGTACTGGTTGGATATG |
| PRDM16 F | CAGCACGGTGAAGCCATTC |
| PRDM16 R | GCGTGCATCCGCTTGTG |
| ELVOL3 F | GATGGTTCTGGGCACCATCTT |
| ELVOL3 R | CGTTGTTGTGTGGCATCCTT |
| DIO2 F | CAGTGTGGTGCACGTCTCCAATC |
| DIO2 R | TGAACCAAAGTTGACCACCAG |
| COX8B F | GAACCATGAAGCCAACGACT |
| COX8B R | GCGAAGTTCACAGTGGTTCC |
| CIDEA F | TGCTCTTCTGTATCGCCCAGT |
| CIDEA R | GCCGTGTTAAGGAATCTGCTG |
| CD137 F | CGTGCAGAACTCCTGTGATAAC |
| CD137 R | GTCCACCTATGCTGGAGAAGG |
| TBX1 F | GGCAGGCAGACGAATGTTC |
| TBX1 R | TTGTCATCTACGGGCACAAAG |
| TMEM26 F | ACCCTGTCATCCCACAGAG |
| TMEM26 R | TGTTTGGTGGAGTCCTAAGGTC |
| CPT1B F | TTCAACACTACACGCATCCC |
| CPT1B R | GCCCTCATAGAGCCAGACC |
| CYT C F | GCAAGCATAAGACTGGACCAAA |
| CYT C R | TTGTTGGCATCTGTGTAAGAGAATC |
| TFAM F | GCTGACGACTTCGACGACG |
| TFAM R | TCGGTCAACAGGAGGTTGTCT |
| SCD1 F | TTCTTGCGATACACTCTGGTGC |
| SCD1 R | CGGGATTGAATGTTCTTGTCGT |
| ACC1 F | TGGACAGACTGATCGCAGAGAAAG |
| ACC1 R | TGGAGAGCCCCACACACA |
| FASN F | AAGCGGTCTGGAAAGCTGAA |
| FASN R | AGGCTGGGTTGATACCTCCA |
| ME F | GACCCGCATCTCAACAAGGA |
| ME R | CAGGAGATACCTGTCGAAGTCA |
| CD36 F | ATGGGCTGTGATCGGAACTG |
| CD36 R | GTCTTCCCAATAAGCATGTCTCC |
| ATGL F | TGTGGCCTCATTCCTCCTAC |
| ATGL R | TCGTGGATGTTGGTGGAGCT |
| MGL F | CTAATTTCACCTCTGATCCT |
| MGL R | AGGACAGAGTTGGTCACTTC |
| HSL F | GCTGGGCTGTCAAGCACTGT |
| HSL R | GTAACTGGGTAGGCTGCCAT |
| LPL F | GGGAGTTTGGCTCCAGAGTTT |
| LPL R | TGTGTCTTCAGGGGTCCTTAG |
| CIDEC F | ATGGACTACGCCATGAAGTCT |
| CIDEC R | CGGTGCTAACACGACAGGG |
| FOXO1 F | TCAAGGATAAGGGCGACAGC |
| FOXO1 R | TGTCCATGGACGCAGCTCTT |
| PPARα F | CAGGAGAGCAGGGATTTGCA |
| PPARα R | CCTACGCTCAGCCCTCTTCAT |
| VLCAD F | CTACTGTGCTTCAGGGACAAC |
| VLCAD R | CAAAGGACTTCGATTCTGCCC |
| UCP-2 F | CGTCTCCCAGCCATTTTCTA |
| UCP-2 R | AGAAACGGGGACCTTCAATC |
| UCP-3 F | CTGCACCGCCAGATGAGTTT |
| UCP-3 R | ATCATGGCTTGAAATCGGACC |
| PDK4 F | AGGGAGGTCGAGCTGTTCTC |
| PDK4 R | GGAGTGTTCACTAAGCGGTCA |
| COX2 F | CCAGAGCAGAGAGATGAAATACCA |
| COX2 R | GCAGGGCGGGATACAGTTC |
| ARG-1 F | CTCCAAGCCAAAGTCCTTAGAG |
| ARG-1 R | AGGAGCTGTCATTAGGGACATC |
| MRC-1 F | CTCTGTTCAGCTATTGGACGC |
| MRC-1 R | CGGAATTTCTGGGATTCAGCTTC |
| FIZZ-1 F | CCAATCCAGCTAACTATCCCTCC |
| FIZZ-1 R | ACCCAGTAGCAGTCATCCCA |
| YM-1 F | CAGGTCTGGCAATTCTTCTGAA |
| YM-1 R | GTCTTGCTCATGTGTGTAAGTGA |
| IFN-γ F | ATGAACGCTACACACTGCATC |
| IFN-γ R | CCATCCTTTTGCCAGTTCCTC |
| CCL-2 F | TTAAAAACCTGGATCGGAACCAA |
| CCL-2 R | GCATTAGCTTCAGATTTACGGGT |
| MB F | CACCATGGGGCTCAGTGATG |
| MB R | CTCAGCCCTGGAAGCCTAGC |
| MHC I F | CTGCCTCTGCACCCCATAATG |
| MHC I R | TTGCTGAGATGACAGAACGCT |
| MHC IIa F | CAGCTGCACCTTCTCGTTTG |
| MHC IIa R | CCCGAAAACGGCCATCT |
| MHC IIb F | CAATCAGGAACCTTCGGAACAC |
| MHC IIb R | GTCCTGGCCTCTGAGAGCAT |
| MHC IIX F | GGACCCACGGTCGAAGTTG |
| MHC IIXR | CCCGAAAACGGCCATCT |
| TNNI1 F | CTGCAGGTTGTGCTAGATGGGATGG |
| TNNI1 R | GCCTGGGGTCTTTGGTAAGTAGGC |
| mitDNA F | CCTATCACCCTTGCCATCAT |
| mitDNA R | GAGGCTGTTGCTTGTGTGAC |
| nuDNA F | ATGGAAAGCCTGCCATCATG |
| nuDNA R | TCCTTGTTGTTCAGCATCAC |
| p38α fragment F | GAAGCGCGAGCGGGTGTCTT |
| p38α fragment R | CGGTAGGTCCTTTTGGCGTG |
| p38β fragment F | CTCGCTCCAGCTGCTTCTGT |
| p38β fragment R | TCGCTGAAATCCTCGATGGA |
| p38γ fragment F | GTATCGGGCACCAGAGGTCA |
| p38γ fragment R | CCCGAAGGGACTCAAAGTAT |
| p38δ fragment F | CGGGATGAGCCTCACTCGGAAA |
| p38δ fragment R | TTTCAGGTCCCTGTGGACGATGCC |
| p38α F | ACAAGACCATCTGGGAGGTG |
| p38α R | GTCCTTTTGGCGTGAATGAT |
| p38β F | GCGGGATTCTACCGGCAAG |
| p38β R | GAGCAGACTGAGCCGTAGG |
| p38γ F | ATCACAGGGACGCCCCCTCC |
| p38γ R | TTCACAGCCTGAGGGCTTGCG |
| p38δ F | ATGAGCCTCACTCGGAAAAGG |
| p38δ R | GCATGTGCTTCAAGAGCAGAA |
