# Supplementary material for: Metabolic benefits of inhibition of p38α in white adipose tissue in obesity
Source: PLoS Biol. 2018 May 11;16(5):e2004225. doi: 10.1371/journal.pbio.2004225 (PMC5965899; doi:10.1371/journal.pbio.2004225)
Supplement: S2 Table — (DOCX) [file pbio.2004225.s009.docx]

**S2 Table. Primary Antibodies**

| p38α | CST 2371S |
| --- | --- |
| p-p38(T180/T182) | CST 4511S |
| p38β | CST 2339S |
| p38γ | CST 2307S |
| UCP-1 | Abcam ab10983 |
| p-ATF2(Thr71) | CST 9221S |
| Tyrosine Hydroxylase(TH) | Merck / millipore AB152 |
| p-CREB(Ser133) | CST 9198S |
| CREB | Abcam ab31387 |
| PKA Cα | CST 4782S |
| P-PKA C(Thr197) | CST 4781S |
| p-PKA substrate (RRXS*/T*) | CST 9624S |
| Perilipin (D1D8)XP | CST 9349S |
| Hsp90 | CST 4874S |
| Tubulin | Sigma T6199-200UL |
| F4/80 | eBioscience 11-4801-82 |
| Mac1 | eBioscience 17-0112-82 |
| Gr-1 | eBioscience 12-5931-82 |
| CD68 | BioLegend 137001 |
| BrdU | Santa Cruz sc-32323 |
